# Evolution and diversity of alpha-carbonic anhydrases in the mantle of the Mediterranean mussel (*Mytilus galloprovincialis*)

**DOI:** 10.1038/s41598-019-46913-2

**Published:** 2019-07-18

**Authors:** João C. R. Cardoso, Vinicius Ferreira, Xushuai Zhang, Liliana Anjos, Rute C. Félix, Frederico M. Batista, Deborah M. Power

**Affiliations:** 1 Comparative Endocrinology and Integrative Biology, Centre of Marine Sciences, Universidade do Algarve, Campus de Gambelas, 8005-139 Faro, Portugal; 2 International Research Center for Marine Biosciences, Ministry of Science and Technology, Shanghai Ocean University, Shanghai, China; 3 Key Laboratory of Exploration and Utilization of Aquatic Genetic Resources, Ministry of Education, Shanghai Ocean University, Shanghai, China; 4 Present Address: Centre for Environment Fisheries and Aquaculture Science (CEFAS), Weymouth, Dorset UK

**Keywords:** Phylogeny, Enzymes, Molecular evolution

## Abstract

The α-carbonic anhydrases (α-CAs) are a large and ancient group of metazoan-specific enzymes. They generate bicarbonate from metabolic carbon dioxide and through calcium carbonate crystal formation play a key role in the regulation of mineralized structures. To better understand how α-CAs contribute to shell mineralization in the marine Mediterranean mussel (*Mytilus galloprovincialis*) we characterized them in the mantle. Phylogenetic analysis revealed that mollusc α-CA evolution was affected by lineage and species-specific events. Ten α-CAs were found in the Mediterranean mussel mantle and the most abundant form was named, MgNACR, as it grouped with oyster nacreins (NACR). Exposure of the Mediterranean mussel to reduced water salinity (18 vs 37 ppt), caused a significant reduction (p < 0.05) in mantle esterase activity and MgNACR transcript abundance (p < 0.05). Protonograms revealed multiple proteins in the mantle with α–CA hydratase activity and mapped to a protein with a similar size to that deduced for monomeric MgNACR. Our data indicate that MgNACR is a major α–CA enzyme in mantle and that by homology with oyster nacreins likely regulates mussel shell production. We propose that species-dependent α-CA evolution may contribute to explain the diversity of bivalve shell structures and their vulnerability to environmental changes.

## Introduction

Bivalves and gastropods are shell-bearing molluscs and the most specious phyla in the marine environment with an important contribution to ecosystem services. The shell is a hard naturally biomineralized structure that supports and protects the soft tissues and stores minerals. The process by which molluscs produce their shell has been studied in many species because they represent an accessible model for studies of biomineralization. Furthermore, there is growing concern that ocean acidification and warming will negatively affect the production of the protective calcified shell and therefore organism survival^1^.

The molluscan shell is predominantly mineralized calcium carbonate crystals (CaCO_3_) in an organic protein matrix (<5% of the shell composition) and its formation depends on the secretory activity of the mantle. Two main mineralized shell structures exist, calcite (prismatic layer) and/or aragonite (nacreous or internal lustrous layer), and the presence of one or other form has been associated with a specific matrix protein composition^2,3^. In the molluscs, the mantle is a large ciliated tissue that coats the inner surface of the shell^4^. The mantle edge is the most active zone of shell deposition and shell growth depends on the availability of calcium (Ca^2+^) and bicarbonate (HCO_3_^−^) obtained from the environment or food^2^. Environmental changes such as modified water salinity affect bivalve shell growth and composition^5–8^. Mantle transcriptomes and mantle and shell proteomes have shed light on candidate shell-forming genes and matrix proteins^4,9–24^. Nonetheless, the understanding of shell production, regulation and repair by the mantle remains largely unresolved and distinct mantle regions appear to contribute differently to this process^11,25,26^.

The carbonic anhydrases (CAs) are a large and ancient group of metalloenzymes common to bacteria, plants and animals. These enzymes accelerate the reversible hydration of metabolic carbon dioxide (CO_2_) to bicarbonate (HCO_3_^−^) a process that requires protons (H^+^)^27–29^ and regulates the formation of the mineralized calcium carbonate crystals in the shell. The CA’s are also involved in other functions including pH regulation, ion-regulation, respiration and photosynthesis^28,30–36^. Five CA superfamilies α-CA, β-CA, γ-CA, δ-CA and ζ–CA that are unrelated in sequence but share similar enzymatic properties have been described and their common activity is ascribed to the three conserved histidine (H) residues that use zinc (Zn^2+^) as a cofactor in their catalytic site^37^. The α-CA family members are restricted to metazoans^28,32^. In vertebrates α-CA’s are classified according to their cellular localization as; cytosolic (CA I, II, III, VII and XIII), membrane-bound (CA IV – glycosylphosphatidyl-inositol (GPI)-linked), transmembrane (CA IX, XII and XIV), mitochondrial (CA VA and VB) and extracellular (CA VI). Another group of proteins related to CAs are the α-CA related-proteins (CARP, that include CA VIII, CA X and CA XI members) that are catalytically inactive and have no assigned biological activity^38^.

In molluscs, nacrein was the first α-CA characterized and was isolated from the nacreous layer of the Japanese pearl oyster (*Pinctada fucata*)^39^. Subsequently, α-CAs were identified in several other tissues including the shell and mantle^15,16,24,40–47^. The availability of molecular data from several molluscs has revealed numerous α-CAs and a complex evolutionary history since multiple gene duplications and speciation events occurred. Recently, a specific group of α-CAs potentially linked to biomineralization was described in molluscs and included forms secreted by the mantle such as nacrein/nacrein-like sequences^28,30^. CA activity can be assessed by measuring the enzymes capacity to release protons during bicarbonate (HCO^3−^) production or by measuring its esterase activity; both mechanisms share the same catalytic pocket^48–51^.

The Mediterranean mussel (*Mytilus galloprovincialis*) is a marine euryhaline bivalve species exploited for aquaculture worldwide. Recently, a unique α–CA, homologous to human α–CA III was purified from the Mediterranean mussel mantle^46^. The identified α–CA was twice the molecular weight of other molluscan α–CAs and had a low capacity to hydrolyse CO_2_, suggesting that it plays a small role in shell mineralization^46^. As the basis for studies to understand shell formation and growth in the Mediterranean mussel, in a previous study we generated several transcriptomes of the mantle edge^11^. Taking into consideration the large number of α–CA’s found in molluscs and the importance of α–CAs in mantle metabolism and shell formation, in the present study we identified them in the Mediterranean mussel mantle transcriptomes. Comparative evolution of the molluscan α–CA family was studied using several non-molluscan species including vertebrates. Their role in the mantle was investigated by characterizing α–CA expression and activity in mussels exposed to full seawater or water with reduced salinity. The mantle is heterogeneous in both function and morphology and in the present study we focused on the posterior mantle edge region which is mainly associated with the growth in length of the mussel shell.

## Materials and Methods

### Animal manipulation and sample preparation

Mediterranean mussel (*M. galloprovincialis*, length 3.76 ± 0.27 cm, wet weight 6.39 ± 1.27 g) were obtained from a local producer in the Ria Formosa (Olhão, Portugal). Mussel mantle edge from the region most distant to the umbo (referred to as the posterior region) was collected from the left valve for RNA extraction and from the right valve for enzymatic assays (esterase and hydratase activities) and tissue histology. For comparative purposes the Pacific oyster (*C. gigas*), for which a sequenced and annotated genome and extensive molecular data exists, and α–CA’s associated with shell formation were used^41^. The oysters (length 3.41 ± 0.27 cm, wet weight 3.71 ± 1.18 g) were donated by Dr. François Hubert (Bivalvia, Olhão, Portugal) and the mantle edge of the flat shell side was collected for hydratase activity assays. Mussels and oysters were transported live to CCMAR and acclimatized for a week in aerated natural sea water (SW) tanks. Animals were anaesthetized in MgCl_2_ (28 mg/L in SW) for 30 min before tissue collection. All tissues were frozen in liquid nitrogen and stored at −80 °C. For histology the mantle was fixed overnight at 4 ^◦^C in 4% paraformaldehyde (PFA).

### α–CA sequence searches

Nucleotide sequences for Mediterranean mussel α–CA (Supplementary Data 1) were retrieved from the mantle transcriptome (SRP 063654)^11^ using local BLAST^52^ with the Pacific oyster homologues^28^ and transcript annotations. The mussel genome (ASM167691v1^53^) was also interrogated but only short sequences were obtained and they were not used for sequence analysis. Homologue sequences from the hard-shelled mussel (*M. coruscus*) were retrieved from an assembled mantle transcriptome (Supplementary Table 1). The mussel sequences were used to identify homologues in the deep-sea vent/seep mussel (*Bathymodiolus platifrons*) and Philippine horse mussel (*Modiolus philippinarum*) genomes^54^. The Pacific oyster, Japanese pearl oyster (*Pinctada funcata*), Eastern oyster (*C. virginica*) genomes and the genomes of two gastropods the owl limpet (*Lottia gigantean*) and California sea hare (*Aplysia californica*) and of the cephalopod octopus (*Octopus bimaculoides*) were also interrogated (Supplementary Table 2). All sequence hits with a cut-off <e-10 were retrieved. The identity of retrieved sequences was confirmed against NCBI (https://www.ncbi.nlm.nih.gov). Human α–CA sequences (15 in total) were used to identify orthologues in chicken (*Gallus gallus*), zebrafish (*Danio rerio*) and an echinoderm (invertebrate deuterostome) the purple sea urchin (*Strongylocentrotus purpuratus*) (Supplementary Table 2).

### Sequence alignments and phylogeny

Sequences were aligned using the MUSCLE algorithm^55^ available in the Aliview platform 1.18^56^. The final alignment (232 sequences and 207 aa positions) was manually inspected to eliminate incomplete and/or highly divergent sequences and gaps (Supplementary Table 2) and was used to construct phylogenetic trees with Maximum Likelihood (ML) and Bayesian Inference (BI) methods. The ML tree was constructed in PhyML 3.0^57^ with SMS automatic model selection according to Akaike Information Criterion (AIC)^58^. The model was a VT substitution model^59^ and the reliability of internal branching was assessed using 100 bootstrap replicates. The BI tree was performed in MrBayes 3.2^60^ using a VT substitution model with 1000000 generations. Both trees were displayed with FigTree 1.4.3 and rooted with the sponge α–CA^28^.

### Experimental challenge

To challenge mussels and assess the effect on α–CA activity we exposed them to an environmental stressor by decreasing the bathing water salinity by half^61–63^. In the experiments mussels were exposed to full SW (control) or diluted SW (brackish water, BW) with (SWF or BWF) or without feeding (SW or BW) for two days and two-weeks. The SW (salinity 37 ppt) was collected from the Ria Formosa and filtered using a Whatman® 0.45 µm filter. BW (salinity 18 ppt) was prepared by diluting (1:1) filtered SW with Elix water and the pH was maintained. Experiments were performed under a natural photoperiod (October 2016, Faro, Portugal) in closed circuit 2L plastic aquariums containing 1L of aerated water at 20 ± 1 °C. Tank water (0.5L) was renewed every 2 days and the pH monitored (8.1 ± 0.1). The mussels that were fed received a fresh microalgae mixture (*Nannochloropsis sp*., *Tetraselmis sp*. and *Isochrysis sp.-* 4.6 × 10^5^ cells/ml) daily. For the two-day challenge, mussels were fed and only exposed to reduced water salinity: SWF (control, n = 12) and BWF (salinity challenged, n = 12). For the groups exposed for two-weeks a feeding challenge was also applied and animals (n = 48 total) were divided into four groups with 12 animals each: SWF (SW and fed), BWF (BW and fed), SW (SW and fast) and BW (BW and fast). All experiments were performed using mussels from the same batch. Mussels were randomly assigned to the different experimental conditions and in both experiments (two-days or two-weeks) three replicate tanks (n = 4/tank) were used per condition. No mortality was observed.

### RNA extraction and cDNA synthesis

Total RNA (tRNA) was extracted from 20–30 mg of tissue with an E.Z.N.A kit (VWR, USA). Tissues were defrosted in lysis buffer and homogenized using a plastic pestle. A DNase I Digestion protocol was performed directly on the columns. For cDNA synthesis, 500 ng of DNAse treated tRNA was used and the reaction was performed with RevertAid-RT (Thermo Fisher, USA) for a 20 μL final volume with 100 pmol random hexamers, 1 mM dNTPs, 200 U of enzyme and 20 U RNase Inhibitor. Reaction conditions were 25 °C, 10 min; 42 °C, 60 min; 70 °C, 10 min. The quality of cDNA was assessed by amplification of the mussel ribosomal subunit *18s* (Table 1) using the following cycle: 95 °C, 3 min; 25 cycles x (95 °C, 20 sec; 62 °C, 20 sec; 72 °C, 20 sec); 72 °C, 5 min.

**Table 1:** List of the primer sequences used in the study.

Name	Sequence (5′-3′)	Annealing Temp. (°C)	Efficiency (%)	R^2^
18S Fwd	*GTGCTAGGGATTGGGGCTTG*	58	99.9	0.99
18S Rev	*TAGTAACGACGGGCGGTGTG*			
Ef1alpha Fwd	*GAAGGCTGAGCGTGAACGTG*	58	100.4	0.99
Ef1alpha Rev	*TCCTGGGGCATCAATAATGG*			
MgCA1 Fwd	*CTCCATTGGTTGTCAAATATG*	58	96.9	0.99
MgCA1 Rev	*ATCGATTGTGTGTTCAGAAC*			
MgNACR Fwd	*AGTGTCAGTGTCCTTCGTTGA*	64	97.3	0.99
MgNACR Rev	*TGCGCAGGTCGTCCAACAT*			

The annealing temperature and the efficiency (%) of the primer pairs and the linearity R^2^ of the standard curve are indicated.

### Quantitative expression

Real-time quantitative PCR (RT-qPCR) was used to determine changes in MgNACR and MgCA1 expression (Table 1). Reactions were performed in duplicate (<5% variation between replicates) using a BioRad CFX Connect Real Time System and SsoFast EvaGreen supermix (Bio-Rad, Portugal). The final reaction volume was 10 µl with 200 nM of both primers and 2 µl of template cDNA (diluted 1:5) in low volume 96-well microplates (Axygen). Optimized cycling conditions were 95 °C, 30 sec; 45 cycles x (95 °C, 5 sec; 58 °C, 10 sec). Melting curves were performed to detect non-specific products and primer dimers. Control reactions were included to confirm the absence of genomic DNA. Elongation factor 1-alpha (*ef1α*) and *18s* (Table 1) were used as the reference genes (cDNA diluted 1:100 and 1:1000, respectively). Data was normalized against the geometric mean of both reference genes.

### Tissue histology

Mussel posterior mantle edge was fixed in 4% PFA for 16 h at 4 °C and was washed in 1x PBS and stored in methanol at −20 °C until tissue processing. Samples were dehydrated through a graded alcohol series (70% to 100%) and embedded in paraffin wax and serial sections (7 μm thick) were cut with a rotary microtome (Leitz, Germany). Sections were mounted on slides coated with 0.01% Poly-L-Lysine and dried at 37 °C overnight and stored at RT until use.

### Enzymatic assays

#### Esterase activity

Carbonic anhydrase esterase activity (n = 6/group) was quantified using a colorimetric assay^64^. Assays were performed in 96 well plates (Greiner, Germany) using mussel posterior mantle edge protein extracts (0.1 mg/µl) prepared in sterile SW. Reactions were performed in duplicate at RT by incubating 10 µl of the extract with 290 µl of the substrate (0.05 M 4-Nitrophenyl acetate (Acros Organics, USA) in Tris-HCl (pH 7.4) for 20 minutes in the dark with gentle agitation. The reaction was stopped by placing it on ice for 5 min and the absorbance was read at 405 nm (Biotek Synergy 4, USA). The amount of p-nitrophenolate produced was quantified using a standard curve of p-nitrophenolate (from 0 to 200 µM). Bovine CA isoenzyme II (BCA II, 0.1 mg/ml) from erythrocytes (Sigma-Aldrich) was used as a positive control. Esterase activity was also measured in the presence of Acetazolamide (AZ, 1 mM, 0.1 mM and 0.05 mM) a specific inhibitor of α–CA^65,66^.

#### Protonography on SDS-PAGE gel

A protonography assay using non-denaturing SDS-PAGE was performed^46,67^. Mussel posterior mantle edge protein extracts (0.1 mg/µl SW, n = 3) were centrifuged at 12,000 rpm 4 °C, 10 min and total protein determined using a Bradford assay with a BSA standard set (Quick Start^TM^, BioRad, USA). For comparison Pacific oyster (*Cg*) mantle protein extracts were prepared using the same procedure. Approximately 1 mg of total mantle protein extract (mussel or oyster) or 0.5 µg of BCA II (positive control) was resolved by 12% SDS-PAGE following the Laemmli method^68^, with the exception that the protein extracts were mixed with Laemmli loading buffer without any reducing agents and were not heated. The electrophoresis was run at a constant current (25 mA) until the dye front ran off the gel.

SDS-PAGE gels were washed with a 2.5% Triton X-100/Tris-HCl (pH 7.4) for 1 h and subsequently washed twice for 10 min in 100 mM Tris-HCl (pH 7.4)/10% isopropanol. The gel was incubated at 4 °C in 0.1% bromothymol blue (BTB, AcrosOrganic, USA) dissolved in 100 mM Tris-HCl (pH 7.4) for 30 min and immediately immersed for 15 min in acidified ddH_2_O (saturated with CO_2_, pH = 4.4–4.6) at RT. α–CA activity was evident as a yellow product on the gel. Negative controls included mantle extracts containing DTT or heat treated (5 min, 100 °C) or the development of gels in non-acidified water (CO_2_ omitted). Images were captured using a SYBR green filter (Chemidoc XRS, Biorad, USA).

#### Protonography on tissue sections

The distribution of α–CA activity was assessed in the mussel posterior mantle edge using an adaptation of the protonography method^46,67^. Tissue sections were dewaxed in xylene and rehydrated (100% to 70% and then water) and incubated for 30 min in 0.1% BTB/100 mM Tris (pH 7.4) at RT. Sections were immersed for 15 min in acidified CO_2_-saturated ddH_2_O. Negative controls included colour development in non-acidified ddH_2_O for 15 min or reactions omitting 0.1% BTB. None of the negative controls gave a colour reaction. Photographs were taken using a microscope (Leica DM2000) coupled to a digital camera (Leica DFC480).

### Statistical analysis

Statistical differences for the esterase enzyme activity assay were detected using a One-Way ANOVA and a Tukey’s multiple comparisons test. The results of the quantitative PCR analysis were analysed using a Mann Whitney test (two-tail, confidence level 95%). The significance cut-off was taken at p < 0.05. Analysis was performed with Prism GraphPad software (7.0).

## Results

### α–CA in molluscs and nomenclature

Sequence searches suggested that the number of α–CAs in molluscs is different in each species. In the Mediterranean mussel mantle edge transcriptome 10 putative α–CA transcripts were retrieved (Table 2) and searches in the Mediterranean mussel genome identified the respective genes as well as additional putative α–CA genes but these sequences were not included in the phylogenetic analysis as they were very incomplete. Searches in the hard-shelled mussel mantle transcriptome identified 20 putative α–CA transcripts (Supplementary Table 1). In the deep-sea vent/seep mussel (*Bathymodiolus platifrons*) and the Philippine horse mussel (*Modiolus philippinarum*) 22 and 34 putative α–CA genes were found, respectively.

**Table 2:** List of the Mediterranean mussel α-CA transcripts expressed in the mantle edge.

Name	Length (aa)	Mantle regions (FPKM)			Homolog	Species	e-value	MW (kDa)	Domains
		Posterior	Middle	Umbo					
**MgNACR**	371	25786,03	16398,91	5723,28	Nacrein-like protein	*M. coruscus*	0.0	43.30	SP, CA
**MgCA1**	306	8653,69	6217,51	2489,02	Carbonic anhydrase 2	*C. gigas*	1e^−76^	34.76	CA
MgCA2^+^	256	1439,84	1342,76	1319,51	Carbonic anhydrase II	*M. galloprovincialis*	0.0	28.41	CA
MgCA3*	191	1134,67	631,81	1588,33	Putative carbonic anhydrase	*M. edulis*	2e^−48^	21.85	CA
MgCA4	403	1012,71	650,70	369,32	Carbonic anhydrase-like protein	*M. coruscus*	1e^−120^	45.47	SP, CA
MgCA5	305	944,05	784,02	1266,06	Carbonic anhydrase 2	*C. gigas*	9e^−79^	34.07	SP, CA
MgCA6*	139	151,29	192,15	328,11	Putative carbonic anhydrase	*M. edulis*	1e^−98^	15.59	CA
MgCA7	319	61,79	45,28	112,71	Carbonic anhydrase 2-like isoform X2	*C. virginica*	4e^−118^	36.56	SP, CA
MgCA8	339	46,34	71,49	49,59	Carbonic anhydrase-related protein-like	*C. virginica*	1e^−118^	39.10	CA
MgCARP	310	47,25	53,62	59,51	Putative carbonic anhydrase-like protein 2 isoform X2	*C. gigas*	1e^−93^	35.48	SP, CA

The length of the mussel α-CA ORF (aa) and their relative abundance (FPKM) in the three transcriptomes for different mantle regions (posterior, middle and umbo) is given^11^. The homology between α-CA transcripts and other mollusc forms and protein predicted molecular weights (MW, kDa) and domains (identified using SMART and SignalP programmes) are also indicated. Mussel α-CAs were named according to sequence similarity and abundance (CA1 to CA8) in the mantle transcriptome. The MgNACR and MgCA1 (most abundant forms) are highlighted in bold. *Incomplete sequences.

*Incomplete. ^+^described in Perfetto *et al*.^46^.

In other bivalves, such as oysters, 26 putative α–CA genes were identified in the Pacific oyster (*C. gigas*) genome but only 8 genes were retrieved from the pearl oyster (*Pinctata fucata*) genome. Analysis of oyster expression data retrieved 10 α–CA transcripts for *P. maxima* and 41 transcripts from the Eastern oyster (*C. virginica*). In the gastropods, the California sea hare (*Aplysia californica*) and Owl limpet (*Lottia gigantea*) genomes 14 and 17 putative α–CA genes were found, respectively. In the cephalopod genome, the octopus (*Octopus bimaculoides*), at least 10 α–CA genes were identified.

The nomenclature attributed to the Mediterranean mussel α–CAs in the present study was based on their sequence similarity with other CAs (Table 2). The Mediterranean mussel (designated Mg from *M. galloprovincialis*) nacrein-like α–CA (MgNACR) was named based on the high sequence similarity with the hard-shelled mussel homologue (AKI87981.1) and nacrein-like proteins from oysters. The Mediterranean mussel MgCARP was named based on its high sequence similarity with the oyster CARP. A nomenclature convention has yet to be established for members of the α–CA family in invertebrates. Thus, we have only annotated the nacrein/nacrein-like members and CARP and the other mussel α–CA sequences are numbered (CA1–8, Table 2). The identified hard-shelled mussel α-CAs were named based on their sequence homology with the Mediterranean mussel sequences (Supplementary Table 1). Due to the large diversity of sequences for α-CAs in molluscs it was not possible to establish a consistent naming system for the different species used in the analysis. The correspondence between the names adopted and the accession numbers is indicated in Supplementary Table 2.

### Phylogeny of the mollusc α–CA

**Figure 1:**
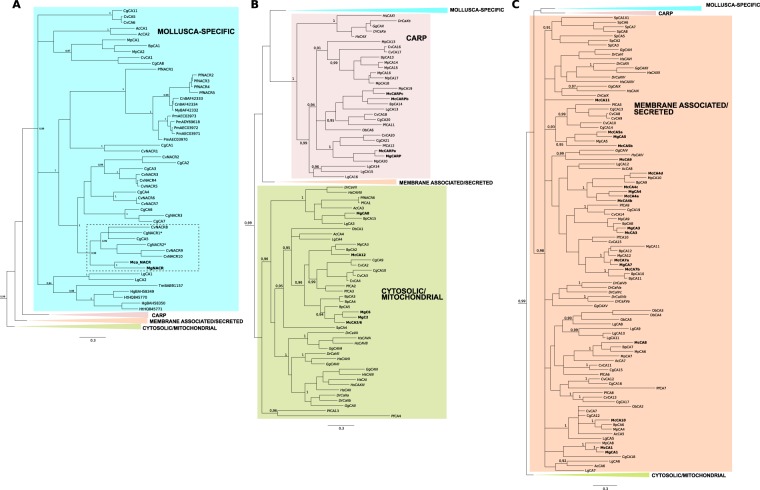
Phylogenetic analysis of the mussel and other metazoan α-CA. The phylogenetic tree was constructed using Bayesian inference (BI) and built in MrBayes 3.2 and branch support values (posterior probability values) are shown. Three subsets of the same phylogenetic tree showing the expansion of the different family members (**A**) (Mollusca-specific, blue), (**B**) (CARP, pink and Cytosolic/Mitochondrial, green) and (**C**) (Membrane associated/Secreted, orange) are represented to facilitate interpretation of the four major α-CA clusters. The Mediterranean mussel (Mg) and hard-shelled mussel (Mc) sequences are highlighted in bold and the mussel cluster is indicated with a dashed box. The oyster (Cg) sequences are indicated with “*” and have been shown to be involved in shell formation^41^. The tree was rooted with the sponge α-CA members (data not shown). The posterior probability values >0.90 at major branches are shown. Description of sequence abbreviations and accession numbers are in Supplementary Table 2. A similar tree was obtained with the ML method (Supplementary Figure 1).

Phylogenetic analysis of the mollusc and other metazoan α–CAs with both BI and ML methods produced similar tree topologies and suggested that they shared common ancestry. Gene duplications occurred prior to the protostome-deuterostome divergence and generated four main α–CA clusters: cytosolic/mitochondrial, membrane associated/secreted, CARP and a Molluscan-specific cluster that includes nacrein and nacrein-like proteins involved in shell biomineralization (Fig. 1A–C)^28^. The majority of the mollusc α-CAs formed a cluster with the vertebrate sequences and the pattern of distribution of the bivalve α–CAs within the different clusters revealed that many members emerged from lineage and species-specific gene duplication events suggesting that different evolutionary pressures shaped α–CA evolution in oysters and mussels.

The Mediterranean mussel mantle α–CA sequences grouped in proximity with the hard-shelled mussel homologues due to the proximate phylogenetic relationship of the species and members were found in each of the four metazoan clusters (Fig. 1A–C). Within the Mollusca-specific cluster only a single α-CA sequence from the Mediterranean (MgNACR) and hard-shelled (McNACR) mussels was found which grouped with the oyster α–CA nacrein/nacrein-like proteins (Fig. 1A). The Philippine horse mussel and the deep-sea vent/seep mussel contained two and one form, respectively within the Mollusca-specific cluster and they were on a separate branch from the mussel NACRs.

In contrast, several oyster α–CAs grouped within the Mollusca-specific cluster suggesting that a large expansion of this gene family occurred (Fig. 1A). In addition, a specific branch for the gastropod α–CAs was found and included the sequences from the green turban (*Turbo marmoratus*), the giant abalone (*Haliotis gigantea*), the green ormer (*Haliotis tuberculata*) and the owl limpet. The separate clustering of the gastropod and bivalve nacrein and nacrein-like α–CAs suggests they evolved differently. Notably, the gastropod, California sea hare, which possesses an internal crystalized shell, had two putative sequences that clustered outside of the gastropod and bivalve clusters, suggesting that specific gastropod nacrein and nacrein protein types may exist. No cephalopod sequence was found within this group.

Several bivalve, gastropod and cephalopod α–CA enzymes including a single transcript from the Mediterranean mussel (MgCARP) and three transcripts from the hard-shelled mussel grouped within the CARP cluster that includes forms of metazoan α–CA lacking the anhydrase esterase activity in vertebrates (Fig. 1B). Three Mediterranean mussel sequences (MgCA2, MgCA6, MgCA8) and two from the hard-shelled mussel, grouped within the cytosolic/mitochondrial cluster (Fig. 1B) but the majority fell within the membrane associated/secreted α–CA cluster (Fig. 1C).

### Sequence comparisons

Comparative sequence analysis between vertebrates and invertebrates identified seven conserved consensus domains (from 4 to 8 aa) that contain the residues essential for the α–CA catalytic activity and structure: domains II and IV contained the three conserved H residues, that bind to the cofactor Zn^2+^ crucial for catalysis; domains III and VI contained the gate-keeping residues: Glutamate (E) in domain III and the first Threonine (T) in domain VI) that orientate the substrate for catalysis^29,69,70^; and domains I, V and VII that are suggested to play an important role in enzyme conformation^28^.

Analysis of the deduced proteins of the Mediterranean mussel α–CAs revealed low sequence conservation and the percent identity was lower than 45% with the exception of MgCA2 and MgCA6 that shared the greatest identity (76% aa). Domains I and III were highly conserved and domain V was the most degenerate and some mussel α–CAs have amino acid mutations within the domains important for enzyme activity and structure. The three catalytic H’s (two in domain II and one in domain IV) were conserved in MgCA1, MgCA2, MgCA5 and MgCA7 but in MgNACR only the first H residue was maintained, and the others were replaced by Glutamine (Q) (Supplementary Figure 2). The position of the gate-keeping residues (domains III and VI) were conserved in all mussel α–CAs. Membrane associated/secreted α–CA members contained a predicted signal peptide except for MgCA1 suggesting that they are secreted proteins. A signal peptide sequence was also predicted for MgNACR and MgCARP (Supplementary Figure 2). The MgCA3 and MgCA6 were incomplete and lacked the N-terminal part of the protein (domains I, II, III and IV) and were not included in the analysis.

**Figure 2:**
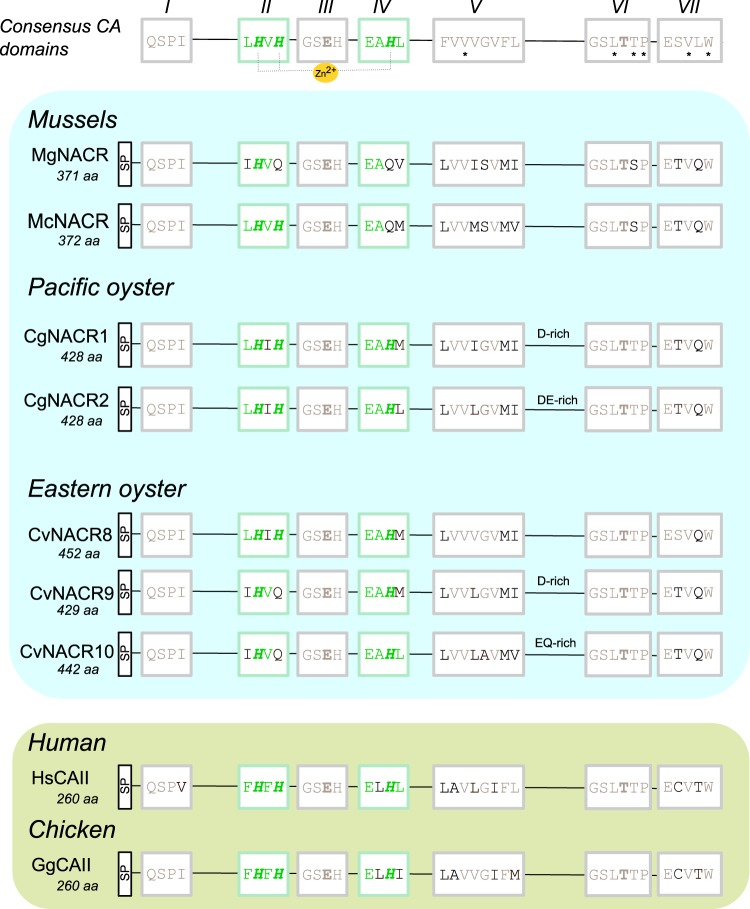
Characterization of the Mediterranean mussel NACR (MgNACR) enzyme catalytic and structural domains. Comparison of the NACR sequence homologues from the hard-shelled mussel (McNACR) and oyster homologues with the human (HsCAII) and chicken (GgCAII) α-CAs involved in osteoclast activity and egg shell biomineralization, respectively. The pacific oyster sequences are involved in shell formation^41^. The seven consensus metazoan domains of α-CAs involved in biomineralization^28^ are indicated. The domains that contain the three histidine (H) residues (highlighted in green and bold italics) that interact with the Zn^2+^ ion within the catalytic site are annotated in green and the domains involved in α-CA conformation are indicated in grey. The gate-keeping residues Glutamate (E) within domain 3 and the first Threonine (T) in domain 6 that orientate the substrate for catalysis are highlighted in grey and bold. The signal peptide (SP) sequence is represented and was predicted using the SignalP 4.1 Server. The predicted size (aa- amino acids) of the deduced proteins is indicated. The residues predicted to delimit the human α-CAs active site cavity are indicated by “*”^71^. The pacific oyster CgCA5 was not included as it is incomplete (lacks domains II and III).

Comparisons of MgNACR with the bivalve α–CA sequence homologues revealed that degeneration of the enzyme catalytic H residues also occurred in the oyster and in hard-shelled mussel NACR sequences (Fig. 2). In the oyster CvNACR9 and CvNACR10 the second H residue (domain II) was replaced by Q and in the hard-shelled mussel McNACR the H residues in domain II were preserved but in domain IV were mutated to Q (Fig. 2). Comparative analysis also revealed that domain V was the most variable however in mussels and oysters only three aa positions were degenerate suggesting that this region evolved differently in bivalves relative to other metazoans. No repetitive amino acid rich region between domain V and VI was found in the mussel sequences.

Sequence comparison of the two mussel NACR with the human and chicken α–CAII (members of the cytosolic/mitochondrial cluster) involved in bone and egg shell mineralization, respectively, revealed that despite the differences in the consensus catalytic sites, other residues important for structure and function were maintained (Fig. 2). The residues that in human α–CAs delineate the catalytic site (within domain V, VI and VII)^71^ were preserved in bivalves suggesting that the protein conformation has been conserved.

### Effect of environmental salinity and starvation

#### Mussel physiology

No significant changes in the length or width of the mussels occurred during the experiments irrespective of treatments. Animal weight and dry shell weight (Supplementary Table 3) at the end of the two week experiment were not significantly different from the control.

### Esterase activity in mantle protein extracts

**Figure 3:**
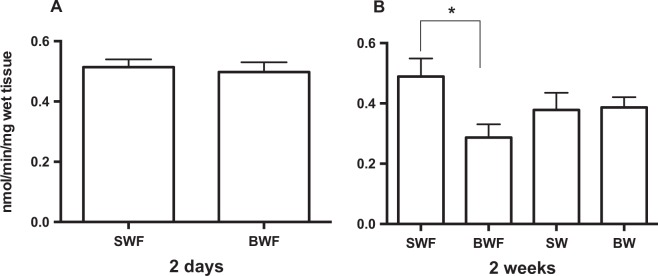
Esterase enzyme activity in the posterior mantle edge of the Mediterranean mussel. Esterase activity was determined using 4-Nitrophenyl acetate as the substrate in mantle protein extracts two days (**A**) and two weeks (**B**) after water salinity was reduced^58^. The results are represented as the mean ± SEM of five to six biological replicates that were performed in duplicate. For the two-week group significant differences were identified using a One-Way ANOVA and a Turkey’s multiple comparisons test. The significance cut-off was taken at p < 0.05. Analysis was performed with Prism GraphPad software (7.0). SWF: seawater fed, BWF: brackish water fed; SW: seawater fast; BW: brackish water fast.

No significant differences were detected between control and BW challenged animals after two days (SW 0.51 ± 0.02 mol/min/mg wet tissue and BW 0.49 ± 0.03 mol/min/mg wet tissue) or between the animals fasted for two weeks in different salinities (SW 0.38 ± 0.12 mol/min/mg wet tissue and BW (0.39 ± 0.07 mol/min/mg wet tissue) (Fig. 3A). In contrast, mantle edge esterase activity after the two week challenge was significantly decreased (*p* < 0.05) in the mussels maintained in BW and fed (BWF 0.28 ± 0.04 mol/min/mg wet tissue) relative to the control maintained in SW and fed (SWF 0.48 ± 0.06 mol/min/mg wet tissue) (Fig. 3B). Enzyme esterase activity in the mantle edge protein extracts was not affected by the α–CA-specific inhibitor acetazolamide (Supplementary Figure 3). In contrast, the activity of BCA II (positive control) was totally inhibited. This suggests that either the mussel α–CA’s are insensitive to the inhibitor acetazolamide or that the activity observed is due to esterase activity of other enzymes.

### Expression in mussel mantle edge

Analysis of the relative transcript abundance (Fragments Per Kilobase of transcript per Million, FPKM) in Mediterranean mussel mantle edge transcriptomes^11^ revealed that the identified α–CAs differed in abundance across the different mantle regions (posterior, middle and umbo regions) (Table 2). MgNACR was the most abundant transcript and gene expression (FPKM) was about 3-fold higher than the second most abundant form (MgCA1) and was predominantly expressed in the posterior mantle region. The MgNACR homologue (McNACR) was also the most abundant transcript (FPKM) in the hard-shelled mussel posterior mantle edge transcriptome (data not shown).

**Figure 4:**
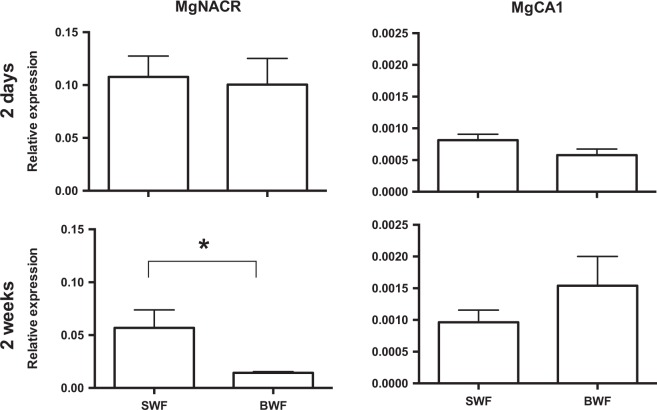
Changes in α-CA gene expression in the posterior mantle edge. Expression of MgNACR and MgCA1 was analysed in the posterior region of the mantle edge in mussels that were exposed for two-days or two-weeks to lower water salinity (BW) or control full seawater group (SW). Gene expression levels were normalized using the geometric mean of two reference genes (*ef1α* and *18s*). The results are represented as mean ± SEM of four to six biological replicates. Prism GraphPad v5 software was used to assess the significance of differences between the experimental groups using a Mann-Whitney (two-tailed) test (*p < 0.05). SWF: seawater fed, BWF: brackish water fed; SW: seawater fast; BW: brackish water fast.

To link the observed changes in enzyme function (Fig. 3) with transcript abundance in the Mediterranean mussel we analysed the expression of the two most abundant transcripts (MgNACR and MgCA1) in the posterior mantle edge of mussel exposed to SW or BW (Fig. 4). No differences in MgNACR and MgCA1 transcript expression occurred between SWF and BWF exposed Mediterranean mussels in the two-days experiment. However, MgNACR transcript expression was significantly lower (p < 0.05) in mussels that were maintained for two weeks in BWF relative to SWF (Fig. 4). No significant changes in expression were observed for MgCA1 after two-days or two-weeks. This indicates that the mussel α–CA genes respond differently to decreased water salinity and that MgNACR is sensitive to environmental water salinity.

### α–CA hydratase activity in mantle protein extracts

**Figure 5:**
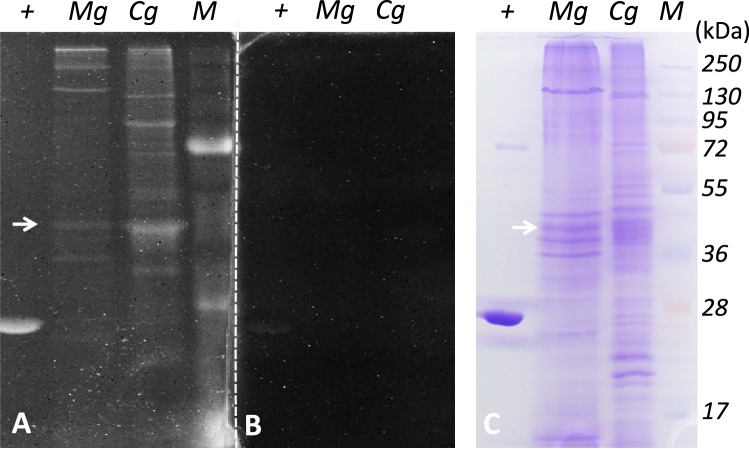
Protonogram and Coomassie blue SDS-PAGE of Mediterranean mussel (*Mg*) and Pacific oyster (*Cg*) mantle protein extracts. (**A**) Protonogram; (**B**) negative protonogram/absence of substrate and (**C**) SDS-PAGE stained with blue Coomassie of native crude protein extracts (≈ 1 mg/well) of the posterior mantle. The positive control (+) corresponded to commercial bovine CA II (BCA II, 5 μg/well). Samples were resolved on a 12% SDS-PAGE gel without reducing agent or thermal denaturation. The white arrows indicate the hydratase activity of the most abundant transcript MgNACR α-CA that has an estimated molecular weight of 43.3 kDa. In oyster protein extracts an intense reaction was observed for a protein of a similar size. M: molecular weight marker (PageRuler Plus, Thermo Scientific).

Positive hydratase activity signals were detected in the protonation assay for several proteins in both mussel and oyster mantle extracts (Fig. 5A). In the mussel, an intense signal occurred in the gel for a protein with the predicted molecular weight of the monomeric MgNACR (≈43.3 kDa) (Fig. 5A,C). Activity was also detected for proteins with a molecular weight greater than 72 kDa (maximum size predicted 45.47 kDa for MgCA4, Table 2) suggesting that mussel CAs can function in an oligomeric state. Positive signals were also detected for smaller α-CA proteins (≈36 kDa) that had a similar molecular weight to that deduced for MgCA2 (Table 2) and with α–CA from bovine erythrocytes (Fig. 5A). The oyster mantle extracts had a similar reaction profile to the mussel mantle although a larger number of proteins reacted (we note that the oyster also has a greater number of α–CA genes than the mussel). Proteins of a similar molecular weight to MgNACR gave an intense signal in oyster and have previously been associated with shell formation in the Pacific oyster (CgNACR1, 49.53 kDa and CgNACR2, 49.8 kDa)^41^ (Fig. 5A).

### α–CA activity in mantle tissue sections

**Figure 6:**
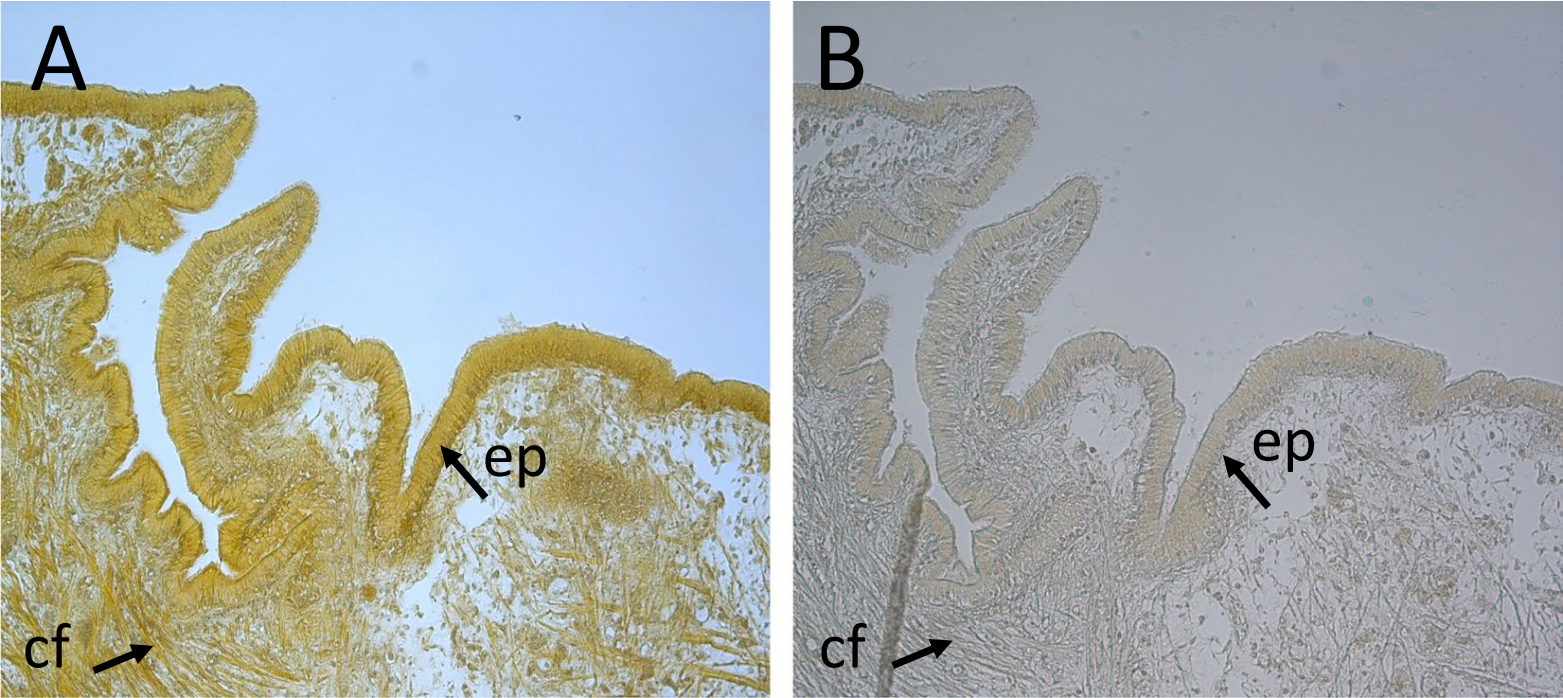
Mapping of α-CA activity in the posterior mantle edge of the mussel. Longitudinal sections of the posterior region of the mantle edge showing α-CA activity in the epithelial cell layer (ep) and in collagen fibres (cf) of the tissue section. A positive reaction (**A**) was observed in tissue sections incubated with 0.1% BTB and acidified with CO_2_-saturated ddH_2_O (pH = 4.4–4.6). The negative control (**B**) corresponds to a tissue section in which 0.1% BTB was omitted from the reaction. No colour reaction was observed. Photographs were taken 15 min after incubation in acidified CO_2_-saturated ddH_2_O (pH = 4.4–4.6) using a digital camera (Leica DFC480) coupled to a Leica DM2000 microscope. Magnification x20.

Hydratase enzyme activity was detected across the mantle indicating that members of the α–CA family have a widespread distribution (Fig. 6A). α–CA activity was evident as intense yellow staining concentrated at the edge of the posterior mantle tissue within the epithelial cells (apparent as a monolayer of homogeneous cells with microvilli). Staining was also observed in the inner mantle region associated with muscle fibers. When acidified water or the substrate was ommitted from the histochemical reaction no colour development occurred (Fig. 6B).

## Discussion

Characterization of mantle α–CAs and their evolution will contribute to understanding mollusc shell production and regulation and the effect of changes in environmental salinity. In this study we describe the α–CA enzyme system in molluscs and specifically the forms that are expressed in the posterior mantle edge of the Mediterranean mussel. Ten α–CA members were found in the mantle edge, a region of intense activity linked to shell growth and comparative analysis of the sequences and expression data suggests that they are involved in different physiological processes. The most abundant α–CA in the mussel mantle was a unique homologue of the bivalve nacrein/nacrein-like protein (MgNACR). This enzyme is structurally similar to other metazoan α–CAs but the catalytic domain that binds to the cofactor Zn^2+^ in vertebrates was mutated in the mussel. Modifying calcium availability by changing the salinity of the bathing water led to a significant reduction in MgNACR transcript abundance and an overall reduction of mantle enzyme esterase activity after two weeks exposure. Protonogram analysis revealed that intense α–CA activity existed in the Mediterranean mussel mantle outer epithelial cell layer and that a protein of a similar molecular weight to the predicted MgNACR protein was abundant and active. Taking into consideration the comparative molecular analysis, tissue localization, abundance, enzyme activity and the response to changed salinity and the previously identified nacrein-like protein in the mussel shell proteome^72^ we propose that the MgNACR identified in the present study may be a potential regulatory factor of mineralization in the Mediterranean mussel and most likely other mytilids.

### Evolution of α–CA in molluscs

α–CAs are an ancient enzyme family and gene duplication has played an important role during their evolution. Distribution of the bivalve α-CAs within the four different clades revealed that they share common ancestry with the deuterostome homologues and that gene duplication occurred prior to the protostome-deuterostome divergence. Independent gene family expansions occurred in both invertebrate and vertebrate lineages as a result of lineage-specific and species-specific events.

In agreement with previous studies^28,73^ a specific cluster that contains bivalve and gastropod α–CAs, named the Mollusca-cluster, was also identified in our study and contained the Mediterranean mussel NACR gene, that clustered with the multiple oyster homologues. The discrepancy in α–CA gene number within the Mollusca-cluster is intriguing and it is tempting to speculate that gene evolution may have been driven by shell diversity. Bivalves have a large diversity of shell forms, shapes and symmetries. The exuberant diversity of shell shapes has been proposed to be explained by the divergent evolution of biomineralization genes in molluscs^23,74–76^. The complex evolutionary profile of α–CAs is reminiscent of what has been observed for the tyrosinases that are another large family of enzymes involved amongst other things in molluscan shell mineralization. Although the tyrosinase genes expanded in oysters, in mussels they, like the CAs, are less numerous^77^.

In mussels and other molluscs, the majority of the identified α-CAs clustered with vertebrate homologues but very few have been functionally characterized. In mammals, cytosolic/mitochondrial α–CA II, III, VB, IX, XII and XIII are associated with cartilage homeostasis and matrix calcification^78^. While membrane bound α–CA IV and *α–*CA XIV are expressed in osteoclasts^78^. In chicken, α–CA II is the main form identified in the egg shell^79–81^. The mussel homologue of human α–CA III^46^ and the oyster homologue of the vertebrate membrane associated/secreted form^40^ have been isolated from the mantle and seem to play a similar role in bivalve shell mineralization to the Mollusca-specific CAs (that includes the NACR gene).

In general, different types of α-CAs are involved in mineralization in molluscs and have a shared origin with the deuterostome homologues. Three major family clusters were established prior to the protostome-deuterostome divergence but the origin of the Mollusca-specific CA cluster is unclear and it may have resulted from a specific gene duplication in the Mollusca lineage or emerged early and was lost from the deuterostome lineage.

### A variety of α–CA are expressed in mussel mantle

The mantle is a tissue common to all molluscs with an important role in shell formation^2,3^. In mussel mantle several α-CAs that in analogy to what occurs in mammals may have distinct cellular localizations (membrane associated, secreted, cytosolic, mitochondrial) and different capacities to catalyse the reaction of bicarbonate (CARP, not functional in vertebrates) were identified. In the mussel posterior mantle edge transcriptome MgNACR and MgCA1 were the most abundant α–CA transcripts and they had a much higher expression in this region than the middle and umbo mantle regions. In contrast, the remaining α–CAs had a similar relative abundance in the three mantle edge regions examined. Identification of a putative signal peptide in MgCA4, MgCA5, MgCA7, MgCARP and MgNACR suggests, based on previous work, that they may be secreted to the shell^24,39,82–84^ while the other α–CAs (MgCA2 and MgCA8) probably remain in the cell cytosol. The grouping of α–CAs by structural diversity was in agreement with the phylogenetic clustering.

Another potential source of functional variability across the mussel α-CA proteins may arise from mutations within the conserved motifs of amino acids crucial for catalytic activity. α-CAs are metalloenzymes and the three conserved histidine (H) residues located in protein domains II and IV that coordinate Zn^2+^ ion binding were conserved in MgCA1, MgCA2, MgCA5 and MgCA7 but mutated in the remaining members. For example, the most abundant transcript in the mantle edge, MgNACR, had only a single conserved H residue within domain II. Nonetheless, substitution of H residues by a glutamine (Q) and asparagine (N) within the Zinc binding site of human α-CA II had no effect on enzyme activity^85^. Furthermore, the enzyme activity measured in mussel mantle protein extracts was similar to that detected in oyster which retains the two H residues in domain II suggesting changes in this domain are not critical for hydratase activity. Mantle enzyme esterase activity was insensitive to acetazolamide (AZ) an α–CA-specific inhibitor. Acetazolamide binds to α–CA and the metal ion at the active site stabilizes the AZ - α-CA complex at the three conserved histidine (H) residues^86^. In MgNACR this catalytic domain is mutated and does not contain the three conserved H residues and this may explain why mussel mantle protein extracts were insensitive to the inhibitor in our study. Oyster recombinant α-CAII enzyme activity is reported to be totally ablated by AZ^66^ suggesting that bivalve α-CAs show differing sensitivities to this chemical.

An interesting observation of our study was the failure of AZ to inhibit mussel and oyster α-CAs and also the positive control (α-CA from bovine erythrocytes) in protonography (data not shown), underlining the importance of α–CA conformation. AZ was also reported to be an ineffective inhibitor of enzyme hydratase activity for the human α-CA III homologue in Mediterranean mussel^46^. The failure of AZ to inhibit α-CAs in mussel mantle meant it was not possible to directly demonstrate the contribution of MgNACR to detected esterase activity and therefore its importance in shell production. Nonetheless, it should be noted that although multiple transcripts for other esterase enzymes were found (carboxylesterase, cholinesterase, phosphodiesterase and others) in the mantle posterior edge transcriptome their relative abundance was low. For example, 125-times and 40-time less that MgNACR and MgCA1, respectively. The preceding observations and the results of protonography suggest that the esterase enzyme activity detected in the mantle protein extract is mostly likely from α–CAs.

The lower number of α-CA transcripts identified in the mantle of the Mediterranean mussel in relation to the hard-shelled mussel was intriguing. Both species belong to the *Mytilus* genus and are phylogenetically proximate, but their shell composition differs, and the Mediterranean mussel possesses a smoother shell^87^, than the hard-shelled mussel. If the differences in shell structure across the bivalves is linked to the number and characteristics of α-CA members in the mantle remains to be evaluated.

### Is mussel nacrein-like α–CA involved in shell regulation?

Despite the large number of α–CA transcripts found in the mussel mantle edge only one transcript, MgNACR, clustered within the Mollusca-clade associated with shell biomineralization. In the posterior mantle edge transcriptome of both the hard-shelled and Mediterranean mussel NACR was by far the most abundant α–CA transcript highlighting its importance in mussel mantle metabolism and its potential involvement in shell formation. Changes in salinity affect bivalve shell calcification and growth^5–8,88^ and ion availability modifies α-CA activity. The results of our study corroborate the results of previous studies since BW caused a significant decrease in MgNACR transcripts and overall tissue esterase activity (an indicator of the α-CA hydratase activity^49^) in the mantle. In contrast, freshwater mussels maintained in distilled water (0 salinity) had increased mantle hydratase activity^89^ but in the pearl mussel *Hyriopsis cumingii* increased calcium availability in water influenced α-CA expression and the higher expression in the posterior mantle pallial was associated with increased nacre deposition in the shell^63^. In the Portuguese oyster *Crassostrea angulata* enzyme activity decreased with lower and higher salinities^90^. When Mediterranean mussels were exposed for 28 days to a hypo- or hypersaline environment (salinity 14 ppt and 38 ppt, pH 7.8, respectively) enzyme hydratase activity in the gills increased slightly relative to the control (pH 7.8, salinity 28 ppt) and the effect was more pronouced when water pH was also decreased^91^ suggesting that α-CA enzymes (transcripts or tissue enzyme activity) are probably more affected by a change in pH than salinity.

α–CA hydratase activity of the posterior mantle edge was associated with a protein of a similar size to that predicted for MgNACR (≈43.3 kDa). However, other proteins with lower and higher molecular weights (probably oligomeric states, as described for other α–CA members) were also observed, indicating that other α–CA family members may also play a role in shell biomineralization. The involvement of multiple α–CAs in shell mineralization is reminiscent of the situation in mammalian bone where multiple α–CAs are involved in mineralization of bone, although one principal form, α–CA II, is most important for osteoclast activity^31,34^. Mussel MgNACR shared similar structural and functional domains with the human and chicken α–CA IIs despite their early divergent evolution from a common ancestral metazoan α–CA gene. We hypothesize that MgNACR, is likely to be a key factor in bivalve shell mineralization and may be the functional equivalent of *α–*CA II.

Mapping of α–CA activity revealed intense staining in the posterior mantle edge epithelial cells that are involved in ion translocation and shell formation and where the calcium ions transported to the shell are concentrated^2,92–94^. The mussel α–CA activity had a similar distribution to nacrein-like α-CA identified by *in situ* hybridization in the epithelia cell layer of the mantle edge in the pearl osyter, *Pinctada fucata*^39,42^. In oysters, α–CA was initially purified from the nacreus-layer of the shell and was suggested to inhibit the crystalization of CaCO_3_ due to the presence of a low complexity domain (LCD) in the C-terminal region consisting of a Gly-x-Asn repeat (where x is any amino acid)^42^. The existence of repeat domains is a characteristic of nacrein and nacrein-like proteins isolated from other congenerous species, *P. maxima* and *P. margaritifera* and from gastropods^28,82^. An LCD domain was absent from the Mediterranean and hard-shelled mussels NACR-like α–CA and the homologue sequences from phylogentically related species of the Mytilidae family. Recently, an α-CA associated with biomineralization was isolated from the Mediterranean mussel mantle and found to be the homologue of human α-CA III. This enzyme was proposed to be dimeric and contained the three conserved H residues within the catalytic site, but its catalytic activity was low making it unlikely to be the shell forming α-CA^46^. The human α-CA III homologue in the Mediterranean mussel corresponded to our MgCA2 transcript and clustered within the cytosolic/mitochondrial cluster in our phylogenetic tree. In our protonogram a low activity α-CA protein with a similar molecular weight (28.41 kDa) to the α-CA positive control was also detected and we propose it may correspond to the enzyme characterized by Perfetto *et al*., 2017.

In summary, in bivalves, α-CAs expanded via lineage-specific and species-specific duplications. The mantle of the Mediterranean mussel expresses a diverse portfolio of α-CAs. However MgNACR, the orthologue of the oyster nacrein/nacrein-like gene (associated with shell mineralization), was by far the most abundant isoform in the mantle and in the posterior edge region where intense shell growth occurs. The direct impact of MgNACR on shell formation remains to be demonstrated but transcript abundance, protein characterization and enzyme activity suggest that this protein may be the principal α-CA involved in mussel shell mineralization. The role of α-CAs in bicarbonate formation and the response of MgNACR to changes in salinity and its presence in mussel shell proteomes^72,95^ makes it a good candidate factor for understanding the impact on shell turnover and growth of changes in the enviroment. The presence in the mussel mantle and most likely other bivalves, of multiple members of the α–CA family is intriguing and presumably creates increased functional versatility the characteristics and scope of which remains to be established.

## Supplementary information

Supplementary Figures, Tables and Data

## Data Availability

All data generated or analysed during this study are included in this published article (and its Supplementary Information files). The datasets analysed during the current study are publicly available and the sources referenced in the text.
