# Supplementary material for: Evolution and diversity of alpha-carbonic anhydrases in the mantle of the Mediterranean mussel (Mytilus galloprovincialis)
Source: Sci Rep. 2019 Jul 18;9:10400. doi: 10.1038/s41598-019-46913-2 (PMC6639325; doi:10.1038/s41598-019-46913-2)
Supplement: Supplementary file 1 — Supplementary Figures, Tables and Data [file 41598_2019_46913_MOESM1_ESM.pdf]

Supplementary Information

Evolution and diversity of alpha-carbonic anhydrases in the mantle of the Mediterranean mussel (*Mytilus galloprovincialis*)

João C.R. Cardoso[1]*, Vinicius Ferreira[1], Xushuai Zhang[1], Liliana Anjos[1], Rute C. Félix[1], Frederico M. Batista[1,4] and Deborah M. Power[1,2,3]*

[1]Comparative Endocrinology and Integrative Biology, Centre of Marine Sciences, Universidade do Algarve, Campus de Gambelas, 8005-139 Faro, Portugal

[2]International Research Center for Marine Biosciences, Ministry of Science and Technology, Shanghai Ocean University, Shanghai, China

[3]Key Laboratory of Exploration and Utilization of Aquatic Genetic Resources, Ministry of Education, Shanghai Ocean University, Shanghai, China

[4]Current address: Centre for Environment Fisheries and Aquaculture Science (CEFAS), Weymouth, Dorset, United Kingdom.

*corresponding authors
JCRC: jccardo@ualg.pt
DMP: dpower@ualg.pt

Running title: α-carbonic anhydrases in mussels

Keywords: α-carbonic anhydrases, bivalves, evolution, mantle, mussel, shell mineralization

Legends

Supplementary Table 1: α -CA transcripts expressed in the hard-shelled mussel (*Mytilus coruscus*) mantle transcriptome. The nomenclature adopted was based on the clustering for the Mediterranean mussel sequences. The predicted size of the largest ORF (aa- amino acids), sequence homologies and predicted domains are indicated.

Supplementary Table 2: Nomenclature and accession numbers of the sequences used for the phylogenetic analysis. *Not used in phylogeny.

Supplementary Table 3: The effect of environmental salinity and starvation on general parameters linked to mussel physiology. The shell length (maximum distance between umbo and the posterior shell tip), height (distance between the two shell valves) and width (greatest distance between the outsides of the closed valves) were measured using a calliper and the whole mussels were weighed (wet weight) at the beginning and end of the two weeks experimental period. Shell weight was recorded from both shells that were washed and dried at 65ºC (minimum of 48 h). SWF: seawater fed, BWF: brackish water fed; SW: seawater fast; BW: brackish water fast

Supplementary Figure 1: Phylogenetic analysis of the mussel and other metazoan α-CA using the ML method. Tree was built using the PhyML software from the ATGC platform. Branch support boostrap values are shown. The sequences of the Mediterranean mussel (Mg) and hard-shelled mussel (Mc) are highlighted in bold. Tree was rooted with the Porifera α-CAs (not shown). Description of sequence abbreviations and corresponding accession numbers are in Supplementary Table 2. Three subsets of the same phylogenetic tree showing the expansion of the different family members (A (Mollusca-specific, blue), B (CARP, pink and Cytosolic/Mitochondrial, green) and C (Membrane associated/Secreted, orange)) are represented to facilitate interpretation of the four major α-CA clusters.

Supplementary Figure 2: α-CA consensus sequence domains in the Mediterranean mussel members. The seven consensus sequence domains characteristic of α-CA involved in biomineralization are represented [25]. The domains with the three catalytic histidine (H) residues (highlighted in green and bold italics) that interact with the Zn^{2+}

ion is annotated in green. The gate-keeping residues within domain III (Glutamate, E) and domain VI (Threonine, T) that orientate substrate for catalysis are highlighted in grey and bold. The signal peptide (SP) sequence is annotated and was predicted using the SignalP 4.1 Server (http://www.cbs.dtu.dk/services/SignalP/). The predicted size (aa-amino acids) of the deduced proteins is also indicated. The residues predicted to delimite the active site cavity of the human α-CAs are indicated by "*" [63]. The mussel transcripts MgCA3 and MgCA6 are not included as they are incomplete (lack domains I, II, III and IV).

Supplementary Figure 3: Effect of acetazolamide (AZ) on esterase enzyme activity in the A) posterior mantle edge of the Mediterranean mussel and B) bovine CAII. Esterase activity was determined using 4-Nitrophenyl acetate as the substrate in the presence or absence (control) of the specific α-CAs inhibitor. The results are represented as the mean ± SEM of five to six biological replicates that were performed in duplicate. Significant differences were identified using a One-Way ANOVA with a Turkey's multiple comparisons test. The significance cut-off was taken at $p < 0.05$. Analysis was performed with Prism GraphPad software (7.0). A) C: control- seawater fed and AZ0.05, AZ0.1 and AZ1 that correspond to the assays performed in the presence of AZ 0.05 mM, 0.1mM and 1 mM, respectively. B) BCAII-bovine CAII and BCA0.05, BCA0.1 and BCA1 that correspond to the assays performed in the presence of AZ 0.05 mM, 0.1mM and 1 mM, respectively.

Supplementary Data 1: α-CA predicted protein sequences from the Mediterranean mussel (*Mytilus galloprovinciallis*) and hard-shelled mussel (*Mytilus coruscus*).

Supplementary Table 1:

Name	Length (aa)	Blastp			Domains
		Homolog	Species	e-value	
McNACR	396	Nacrein-like protein	*M. coruscus*	0	TM, CA
McCA1	313	Carbonic anhydrase 1	*C. gigas*	7E-74	CA
McCA2/6	294	Carbonic anhydrase	*C. gigas*	3E-111	CA, TM
McCA3	341	Carbonic anhydrase 14	*C. gigas*	2E-91	SP, CA
McCA4a	319	Carbonic anhydrase	*M. coruscus*	2E-91	CA
McCA4b	331	Carbonic anhydrase	*M. coruscus*	0	SP, CA
McCA4c	355	Carbonic anhydrase	*M. coruscus*	1E-52	SP, CA
McCA4d	319	Carbonic anhydrase	*M. coruscus*	7E-68	CA
McCA5a	325	Carbonic anhydrase 10	*C. gigas*	3E-93	CA
McCA5b	321	Carbonic anhydrase 10	*C. gigas*	6E-101	TM, CA
McCA7a	287	Carbonic anhydrase	*M. coruscus*	1E-99	SP, CA
McC7b	186	Carbonic anhydrase	*M. coruscus*	5E-51	CA
McCA8	292	Carbonic anhydrase 2	*C. gigas*	3E-63	SP, CA
McCA9	303	Carbonic anhydrase	*C. gigas*	4E-45	SP, CA, TM
McCA10	313	Carbonic anhydrase 2	*C. gigas*	3E-89	SP, CA
McCA11	613	Carbonic anhydrase 2	*C. gigas*	1E-72	CA
McCA12	297	Carbonic anhydrase II	*M. galloprovincialis*	2E-83	TM, CA
McCARPa	448	Carbonic anhydrase	*C. gigas*	1E-105	CA
McCARPb	182	Carbonic anhydrase 1	*C. gigas*	7E-32	CA
McCARPc	257	Carbonic anhydrase II	*M. galloprovincialis*	4E-158	CA

Supplementary Table 2

	Gene	Uniprot/RefSeq	Database
**PROTOSTOMES**			
**MOLLUSCS**			
**Bivalves**			
***Pinctada fucata* (Pf)**			http://marinegenomics.oist.jp ver2.0
PfNACR1	pfu_aug2_0_214_1_13803		
PfNACR2	ni	AMW92814	
PfNACR3	ni	A0ZSF2	
PfNACR4	*ni*	Q27908	
PfNACR5	*ni*	BAA11940	
PfNACR6	pfu_aug2.0_920_1_01244		
PfCA1	*ni*	BAJ52887	
PfCA2	pfu_aug2.0_224.1_13827	BAM75190	
PfCA3	pfu_aug2.0_539.1_30879		
PfCA4	pfu_aug2.0_7459.1_32953		
PfCA5	pfu_aug2.0_1849.1_31939		
PfCA6	pfu_aug2.0_1849.1_31938		
PfCA7	pfu_aug2.0_107.1_23472		
PfCA8	pfu_aug2.0_107.1_23471		
PfCA9	pfu_aug2.0_583.1_10953		
PfCA10	pfu_aug2.0_583.1_10956		
PfCA11	pfu_aug2.0_2182.1_08768		
PfCA12	pfu_aug2.0_551.1_04328		
PfCA13	pfu_aug2.0_374.1.14069		
	ni	AMW92815*	
	pfu_aug2.0_10958.1_29717*		
	pfu_aug2.0_551.1_04327*		
	pfu_aug2.0_8203.1_12978*		
	pfu_aug2.0_4146.1_22513*		
	pfu_aug2.0_1536.1_21678*		
	pfu_aug2.0_1907.1_25196*		
	pfu_aug2.0_1294.1_14936*		
	pfu_aug2.0_1301.1_04951*		
***Crassostrea gigas* (Cg)**			http://metazoa.ensembl.org
CgNACR1	CGI_10025540	KC563207	
CgNACR2	CGI_10014170	KC563208	
CgNACR3	CGI_10027598	EKC34889	
CgCA1	CGI_10018508	EKC36446	
CgCA2	CGI_10016369	EKC34781	
CgCA3	CGI_10026768	EKC41177	
CgCA4	CGI_10013772	EKC35764	
CgCA5	CGI_10028495	EKC41746	
CgCA6	CGI_10027599	EKC34890	
CgCA7	CGI_10001795	EKC22661	
CgCA8	CGI_10011324	EKC18733	
CgCA9	CGI_10009060	EKC31881	
CgCA10	CGI_10009059	EKC31880	
CgCA11	CGI_10020143	EKC41232	
CgCA12	CGI_10017570	EKC37807	
CgCA13	CGI_10010889	EKC32754	
CgCA14	CGI_10006442	EKC23653	
CgCA15	CGI_10004927	EKC34762	
CgCA16	CGI_10004992	EKC20938	
CgCA17	CGI_10025817	EKC26360	

CgCA18	CGI_10017509	EKC32354	
CgCA19	CGI_10014201	EKC33179	
CgCA20	CGI_10025596	EKC40542	
CgCA21	CGI_10007458	EKC19847	
	CGI_10009863*	EKC25721*	
	CGI_10000698*	EKC17496*	
	CGI_10018982*	XP_011419145.1*	
***Crassostrea virginica* (Cv)**			https://blast.ncbi.nlm.nih.gov (taxid:6565)
CvNACR1		XP_022290498	
CvNACR2		XP_022295701	
CvNACR3		XP_022294117	
CvNACR4		XP_022294713	
CvNACR5		XP_022295093	
CvNACR6		XP_022299228	
CvNACR7		XP_022299130	
CvNACR8		XP_022342986	
CvNACR9		XP_022293510	
CvNACR10		XP_022298231	
CvCA1		XP_022305243	
CvCA2		XP_022332513	
CvCA3		XP_022332514	
CvCA4		XP_022331723	
CvCA5		XP_022307359	
CvCA6		XP_022307357	
CvCA7		XP_022341512	
CvCA8		XP_022312365	
CvCA9		XP_022344584	
CvCA10		XP_022337157	
CvCA11		XP_022325476	
CvCA12		XP_022317159	
CvCA13		XP_022317158	
CvCA14		XP_022339695	
CvCA15		XP_022339698	
CvCA16		XP_022328059	
CvCA17		XP_022327820	
CvCA18		XP_022320282	
CvCA19		XP_022320885	
CvCA20		XP_022294978*	
		XP_022294979*	
		XP_022337873*	
		XP_022339700*	
		XP_022339697*	
		XP_022339696*	
		XP_022332515*	
		XP_022332516*	
		XP_022320886*	
		XP_022298247*	
		XP_022294986*	
***Mytilus coruscus* (Mc)**			Transcriptome
McNACR	Mco_c109535_g1		
McCA1	Mco_c86038_g1		
McCA2	Mco_c107700_g1		
McCA3	Mco_c86076_g1		
McCA4	Mco_c80133_g1		
McCA5	Mco_c99436_g1		
McCA6	Mco_c86746_g2		
McCA7	Mco_c108912_g1		
McCA8	Mco_c89015_g1		
McCA9	Mco_c94327_g1		
McCA10	Mco_c96544_g1		
McCA11	Mco_c109076_g1		
McCA12	Mco_c62726_g1		
McCA13	Mco_c88569_g2		
McCA14	Mco_c88569_g1		

McCA15	Mco_c82392_g1	
McCA16	Mco_c107895_g1	
McCA17	Mco_c92789_g1	
McCA18	Mco_c101168_g1	
McCA19	Mco_c107890_g1	
Modiolus philippinarum **(Mp)**		https://blast.ncbi.nlm.nih.gov (GCA_002080025.1)
MpCA1	scaf_39025_0.8	
MpCA2	scaf_39025_0.9	
MpCA3	scaf_51669_0.12	
MpCA4	scaf_69963_0.10	
MpCA5	scaf_26403_0.9	
MpCA6	scaf_35602_0.1	
MpCA7	scaf_19237_0.6	
MpCA8	scaf_26403_0.10	
MpCA9	scaf_62670_0.11	
MpCA10	scaf_66372_0.10	
MpCA11	scaf_55080_0.1	
MpCA12	scaf_25765_0.8	
MpCA13	scaf_13577_3.3	
MpCA14	scaf_3038_0.1	
MpCA15	scaf_3108_0.0	
MpCA16	scaf_50026_0.4	
MpCA17	scaf_3832_0.2	
MpCA18	scaf_62553_0.1	
MpCA19	scaf_25074_1.32	
MpCA20	scaf_34239_0.2	
	scaf_22956-0.4*	
	scaf_69626-1.1*	
	scaf_69626-1.2*	
	scaf_20109-0.2*	
	scaf_3385-0.3*	
	scaf_39151-0.3*	
	scaf_73997-0.1*	
	scaf_20109-0.3*	
	scaf_39151-0.4*	
	scaf_73422-0.2*	
	scaf_63701-0.0*	
	scaf_62553-0.1*	
	scaf_25657-1.3*	
	scaf_5885-0.1*	
Bathymodiolus platiforons **(Bp)**		https://blast.ncbi.nlm.nih.gov (GCA_002080005.1)
BpCA1	scaf_27306-1.27	
BpCA2	scaf_33596-9.4	
BpCA3	scaf_48274-0.3	
BpCA4	scaf_33596-7.3	
BpCA5	scaf_33596-7.4	
BpCA6	scaf_54034-9.8	
BpCA7	scaf_10028-0.14	
BpCA8	scaf_56290-1.11	
BpCA9	scaf_17778-5.14	
BpCA10	scaf_22771-0.36	
BpCA11	scaf_22588-0.14	
BpCA12	scaf_56290-0.6	
BpCA13	scaf_1989-10.5	
BpCA14	scaf_14941-0.4	
BpCA15	scaf_34315-1.12	
	scaf_9415-3.7*	
	scaf_51215-4.6*	
	scaf_5334-6.3*	
	scaf_22771-0.28*	
	scaf_51491-1.2*	
	scaf_62476-1.21*	
	scaf_51491-1.3*	

**Gastropods**			
Aplysia californica **(Ac)**			https://blast.ncbi.nlm.nih.gov (GCF_000002075.1)
AcCA1	scaffold00194	XP_005098882	
AcCA2	scaffold00059	XP_005093230	
AcCA3	scaffold00599	XP_005107445	
AcCA4	scaffold00018	XP_012941697	
AcCA5	scaffold00548	XP_005106718	
AcCA6	scaffold01344	XP_005112694	
AcCA7	scaffold00071	XP_012935478	
AcCA8	scaffold00098	XP_005095301	
	scaffold01234	XP_012945802*	
	scaffold01033	XP_012945196*	
	scaffold00857	XP_012944468*	
	scaffold00100	XP_012936251*	
	scaffold00857	XP_012944467*	
	scaffold00098	XP_005095300*	
Lottia gigantea **(Lg)**			http://metazoa.ensembl.org
LgCA1	LotgiG239188	XP_009052190	
LgCA2	LotgiG238082	XP_009047380	
LgCA3	LotgiG114576	XM_009052754	
LgCA4	LotgiG205401	XP_009052992	
LgCA5	LotgiG131044	XP_009064267	
LgCA6	LotgiG84742	XP_009057610	
LgCA7	LotgiG66515	XP_009053012	
LgCA8	LotgiG126803	XP_009061338	
LgCA9	LotgiG172731	XP_009048147	
LgCA10	LotgiG202179	XP_009053020	
LgCA11	LotgiG239341	XP_009053021	
LgCA12	LotgiG239347	XP_009053029	
LgCA13	LotgiG165569	XP_009060852	
LgCA14	LotgiG124107	XP_009059357	
LgCA15	LotgiG138561	XP_009046954	
LgCA16	LotgiG125950	XP_009060896	
	LotgiG238084*		
**Cephalopod**			
Octopus bimaculoides **(Ob)**			http://metazoa.ensembl.org
ObCA1	Ocbimv22001551m.g	XP_014784758	
ObCA2	Ocbimv22037250m.g	XP_014782844	
ObCA3	Ocbimv22006872m.g	XP_014786584	
ObCA4	Ocbimv22032523m.g	XP_014780512	
ObCA5	Ocbimv22033130m.g	XP_014790542	
ObCA6	Ocbimv22013710m.g	XP_014768996	
	Ocbimv22027531m.g*		
	Ocbimv22016071m.g*		
	Ocbimv22012734m.g*		
	Ocbimv22023623m.g*		
**DEUTEROSTOMES**			
**CEPHALOCHORDATE**			
Strongylocentrotus purpuratus **(Sp)**			http://metazoa.ensembl.org/
SpCA1	SPU_012518	XP_003726289	
SpCA2	SPU_013458	XP_003725538	
SpCA3	SPU_013459	XP_011670431	
SpCA4	SPU_016740	XP_782997	
SpCA5	SPU_025722	XP_011667172	
SpCA6	SPU_001138	XP_796525	
SpCA7	SPU_017421	XP_011681574	
SpCA8	SPU_022346	XP_011679839	
	SPU_004135*		
	SPU_026483*		

	SPU_008658*		
	SPU_026747*		
	SPU_000702*		
	SPU_009509*		
	SPU_008894*		
	SPU_024809*		
	SPU_012995*		
**CHORDATE**			
***Homo sapiens* (Hs)**			
HsCAI		NP_001729	
HsCAII		NP_000058	
HsCAIII		NP_005172	
HsCAIV		NP_000708	
HsCAVA		NP_001730	
HsCAVB		NP_009151	
HsCAVI		NP_001206	
HsCAVII		NP_005173	
HsCAVIII		NP_004047	
HsCAIX		NP_001207	
HsCAX		NP_064563	
HsCAXI		NP_001208	
HsCAXII		NP_001209	
HsCAXIII		NP_940986	
HsCAXIV		NP_036245	
***Gallus gallus* (Gg)**			http://www.ensembl.org
GgCAII	ENSGALG00000030781	NP_990648	
GgCAIII	ENSGALG00000033700	NP_001264340	
GgCAIV	ENSGALG00000005360	XP_415893	
GgCAVA	ENSGALG00000005855	XP_414195	
GgCAVI	ENSGALG00000002390	XP_425745	
GgCAVII	ENSGALG00000033720	XP_414152	
GgCAIX	ENSGALG00000021340	XP_001233320	
GgCAX	ENSGALG00000002993	XP_415644	
GgCAXII	ENSGALG00000003456	XP_413756	
GgCAXV	ENSGALG00000005896	XP_415218	
***Danio rerio* (Dr)**			http://www.ensembl.org
DrCaIIa	ENSDARG00000011166	NP_571185	
DrCaIIb	ENSDARG00000014488	NP_954685	
DrCaIVa	ENSDARG00000043589	NP_001107879	
DrCaIVb	ENSDARG00000042293	NP_001159683	
DrCaIVc	ENSDARG00000044512	F1QK51	
DrCAVA	ENSDARG00000101778	NP_001104671	
DrCaVI	ENSDARG00000056499	E7F742	
DrCaVII	ENSDARG00000045139	NP_957107	
DrCaVIII	ENSDARG00000039098	NP_001017571	
DrCaIX	ENSDARG00000102300	A0A0R4IXD2	
DrCaXa	ENSDARG00000052644	NP_001032198	
DrCaXb	ENSDARG00000009568	XP_696967	
DrCaXII	ENSDARG00000045644	X1WD95	
DrCaXIV	ENSDARG00000061697	NP_001315073	
DrCaXVa	ENSDARG00000015654	NP_001075158	
DrCaXVb	ENSDARG00000040510	NP_998347	

Supplementary Table 3

	SWF	BWF	SW	BW
Length (cm)	4.02 ± 0.37	3.94 ± 0.38	4.20 ± 0.36	4.09 ± 0.24
Width (cm)	2.82 ± 0.18	2.88 ± 0.30	3.01 ± 0.22	3.05 ± 0.14
Weight (g)	7.02 ± 0.87	6.68 ± 1.89	7.39 ± 1.88	7.23 ± 1.27
Shell dry weight	3.0 ± 0.41	3.07 ± 1.03	3.19 ± 0.79	3.24 ± 0.69

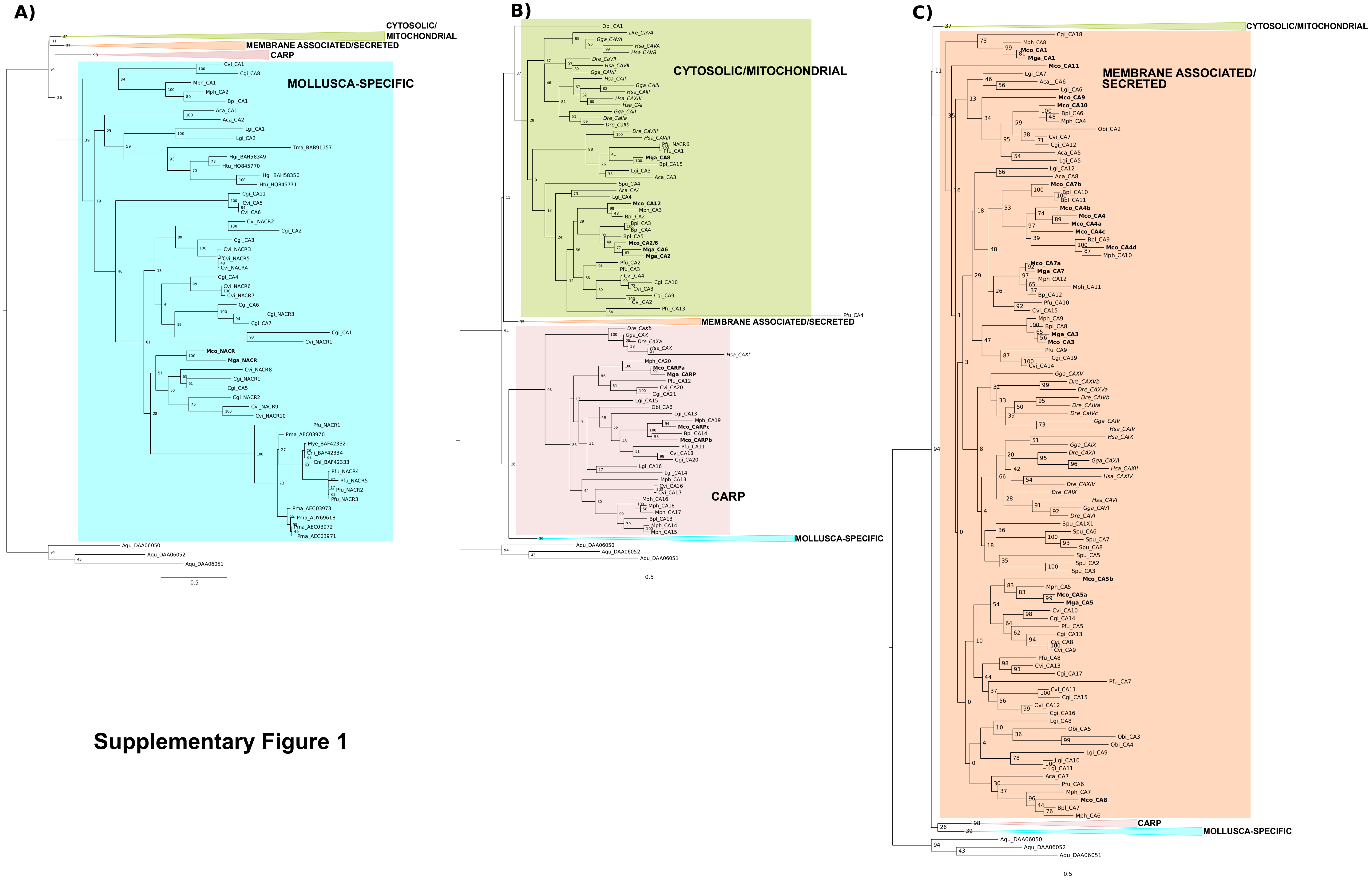

Supplementary Figure 1

Supplementary Figure 2

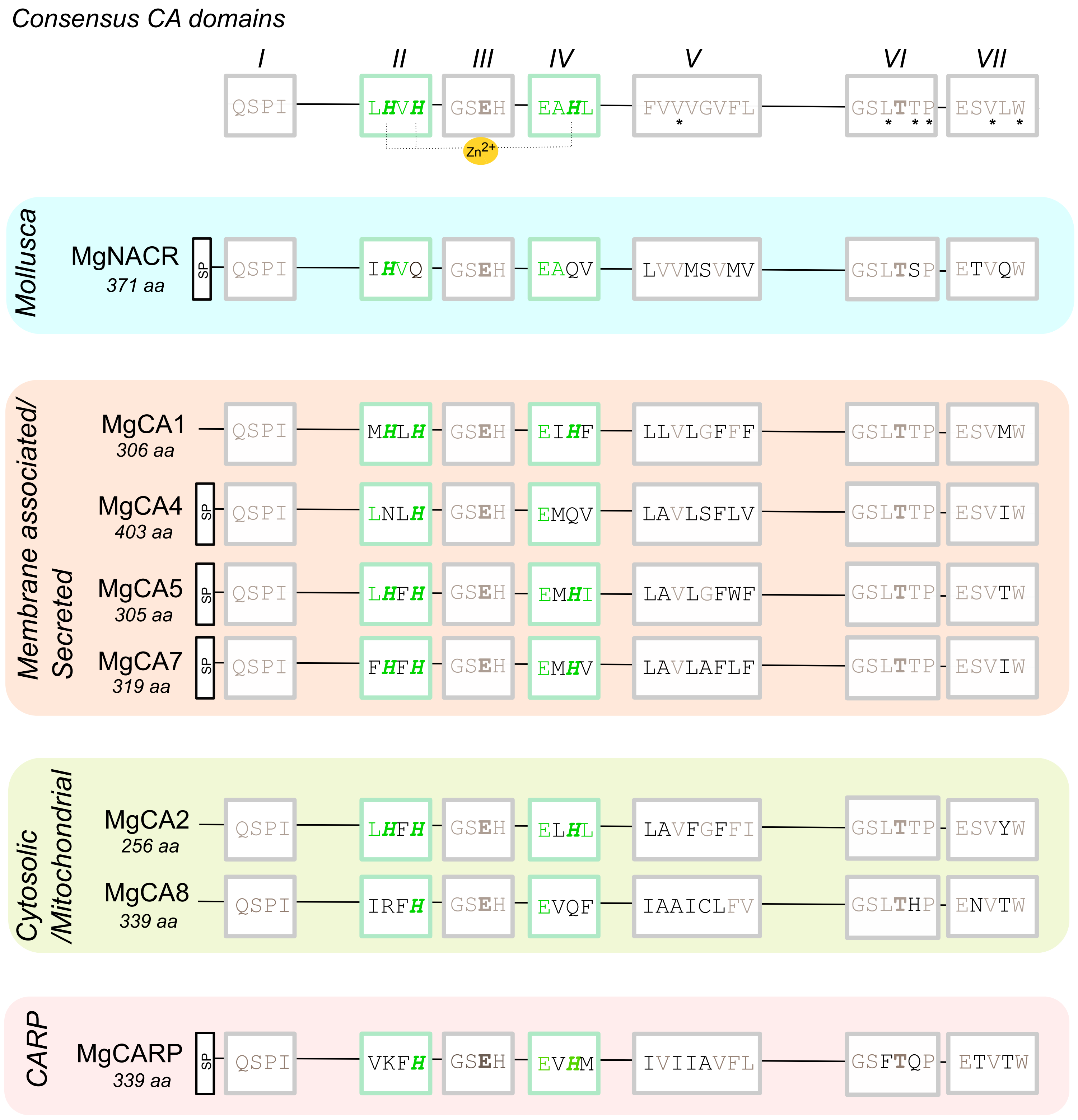

Supplementary Figure 3

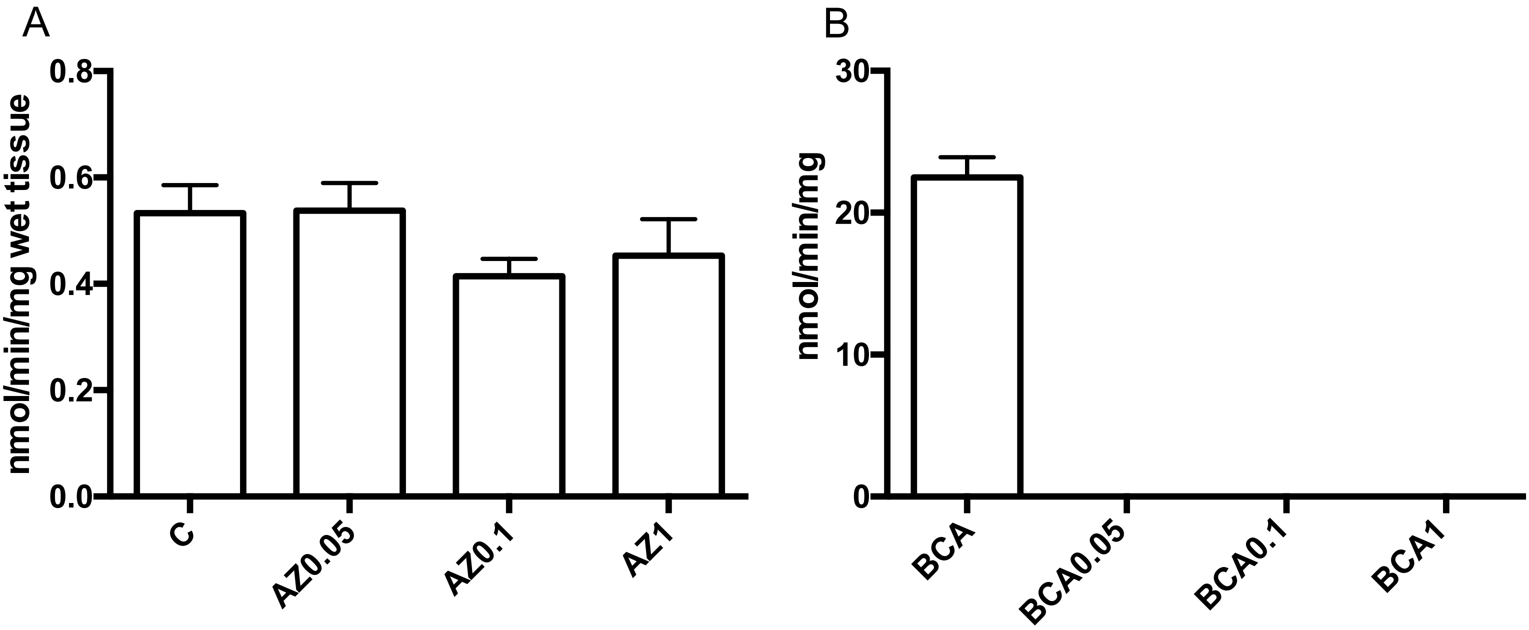

Supplementary Data 1

Mytillus galloprovincialis

>MgNACR
MELTFLCATLAAVSVSFVDGVGYMGVRPPSLKQCCYKDIDKAHFSYDNNLCEGPDNWCK
VHPCWTTCASKKSQSPININTGLTRYKRYPRLKFEKILRRVLANVRNNGHAPYFEVHER
YDDEITVRNVPERPRQKEYNFAQIHVQLGQDEKNGSEHSIDNVFKPMEAQVVFYDKDYE
DVTDAKSKKRGLVVMSVMVEVYGQKPENDACGCDQETCTVRYARRLSALMEKYYEKVRR
YPLASINPDFWTFLSLPKKCWYNRCGKTPTKEFIKNRCEKEEPDRRPFRVFEGITPLDV
LPYDTNRFYTYSGSLTSPPCYETVQWIVYKCPIKVSAKAFKMLQLVEDSHLKPLPQLGT
RRPQQNMNVPVYRSYLK

>MgCA1
MGELSVMLTLSLLYGNIIDPKTGKDQTGHHWSYFGDSGPDHWSDHYPRCNGHRQSPIDI
RTGDVEFASWLTELQFYGYDKNLGQGDPCTIKNNGHTASMAITHSLKLRGAHLNGDYQL
AEMHLHWGHNNSRGSEHALNGKKYPAEIHFVHYSTRYGNLSEAMKMPDGLAVIGVFVEL
VPEDTYAFEQLANGINTILEYGKKANVSNVRMLDFLPQRKNAFYHYRGSLTTPPCYESV
MWFVMADTIPMSERQLDRYRNLQEKVDEHVAATGKASSVSSRVFKSDPIYIRENFRPLQ
PLNGRKVYSNVMPLQRPQLRDFELPAVKVSPIRNNYLPPQAPNRIPVDNSQSSNYHANS
RTDYIYRGLSPNSYNNQRQSSFNGIVSNGNSLLIGNIKPNDINGGLYQHLRPNQQYPRQ
GSPVNKINTGHAFSNGNSERMHVFKTPETASVLSNRRKAGKSGPLLDYIFTQMNGLTKP
NTQNQRHYHANDHPGIAQPKDRIFAGLSDFIANPVQSRVSSNPNNNYIQSSGNGNFNQQ
KLISNQYGNHLQKHFGQSPPSLQNNHGPGNYVQAPVYPDYHQSRNGNSLTVFGYGLS

>MgaCA2
MSWGYGNDNGPCTWCNQFPIANGKRQSPIDICPDKITCDQQLANSPLVVKYEKEPKAEA
MNTGKSVKVQATKASEISGGPLTGTYRLEQFHFHWGADDNKGSEHTIDGKMYAAELHLV
HYNTKYANFGEAVDKPDGLAVFGFFIKPGAKHVGMKELTDNTLCSITAEGKTCNMPCDL
DMASLLNSDLTKYWTYLGSLTTPPLYESVTWIVFQDPIEMSNDQLQALRNLKCGSNFIV
DNYRPPVPLGDRTVRASFKN

>MgCA3
DRFQKIEDAIDKPDGLLVLGFFFEVGRHNIHFEEVFNYLENVAHKGDTTEIPPVIMERL
VPIDLVHYYRYRGSLTTPPCYESVVWTVFTQPITISSSQMSDFRNKIKKTTVNATHEEN
LVDDFRPLQQLNGRNVYSSFVTQKPSTQAPQTVQPINVVCQHSHDNTNNAINTNFSLFV
IFISILTSGYTCGH

>MgCA4
MKFTAAVTSVFFLIGSYFANGAVPEWGYHHGVDPKHWKYLFPTCGGMQQSPIDIKTSKV
QTDITLEHFDLSKLSTAKNVELGIQKDGQTVQVGLKGQGLEVSGGGLPGTYVVEQLNLH
WGSKNTRGSEHEINEKHFSMEMQVVMHTDIFPSVNDAMNCTNGLAVLSFLVDIGPHNHK
FDEIINHLYSIEHIGGHAQLNSFSLTSLFPSTDYFYRYYGSLTTPPCFESVIWTIFKEQ
IYISEFQLNQFRQLGRAHFIGTGDLINNYRFPQPLNHRVVTSNFIQTNTIKTPKPLRVK
ISKSKSGKKSRSRRNRKSKSRSSKKSKSRRGKKSKSTRGKKSKSASGKKSNYRSSKRSN
YRSSKKSKSGSGIKSQSISGLPLLTRRAYSDFNKNLFFRRRYGDSDSSD

>MgCA5
MFYSPTLLVLFGLINDFVTCAEWGYSSPYGPSNWTTLHGNEACDRRAQSPIDIPDAMNA
QYDEGLVPFTVMGFEKKTNQLKLINNGHTAQLNILGDLTVSGGGLSGTFKTAQLHFHWG
SSSIKGSEHLRNTKAFPLEMHIVNFNSKYGNLGTAADKPDGLAVLGFWFKVVAEDNKMF
ARFVSNLTSIQNADAEVDVTDVLVSDLIIPKLDKYYRYKGSLTTPPCFESVTWTMFEET

IPISESQLRQFRNLKEKQPSHNIVNNFRPVQMLNGRRVSRSFKLETDGASTICSSSIIT
VLLVYLLHLL

>MgCA6
LVHYNTKYPNFGAAADKPDGLAVFGFFVKPGAKHVGMKDLTDTAFAAVRDEGKKVHMTS
DLDMKTLLNSDTTKYWTYLGSLTTPPLYESVTWIVFKEPIELSDEQLHELRNLRCGSNC
IVNNYRPPVPLGDRSVRASFK

>MgCA7
MEFLLLSLYQLLVLLYLISNTKITCSISGNKWGYDYADGPKFWPNSYPNCGGQQQSPIS
IGVSEVVVNNSLPQFNFDHIKNVTNLKMTLENNGHSVQIDLKGNNLQLSGGGLPGVYNA
EQFHFHWGSEDKRGSEHNINGKQYPMEMHVVLYNSQHGVFSNALNKTGGLAVLAFLFEI
GETNHHFDEIISHLTKISHKDDHALLNTFALDSLFPHDAGVYYRYYGSLTTPPCYESVI
WTIFKNHIIISEEQIDKFRHQVHRNYANETDRDISDDYRPVQRLNNRVVMTNAEDGLYY
NNCKINLPFNFYFMVLLFLLISKL

>MgCA8
MNFCRNILKKIWHCLRVVCVTQMSYSQSEDVPSRPVKRTNSSSWMINIVRTVRFKHDAL
KDHGFWYLKSSEWSTQFPDSNGTNQSPINLVSKTAVYDPKFKEKLLSISYSSSRETDFL
NNGQTVVIYPKSRNDTSVRQEQAVRSVITGGPLDDDEYELAEIRFHWGRCSRRGSEHTV
NGKAFPMEVQFIHWNSTLYQNLEEATGKENGIAAICLFVQIGRENPAFKALIADSLDDI
LYKGRQKTTYAPFNPASLLPVSELLHDYWVYDGSLTHPPCSENVTWILLRYPLLLSQEQ
AMDFRRLNTYSQETKVTNAYEGKLVDNFRPIQPLNDRKIRASFQ

>MgCARP
MSWMVLFALILPKSFANELGKVWGYDSHLAPEYWGKLHGKEWDLCKSGKHQSPIEIDPS
LLLFDPNLTNLHINTANVNGRLETNGLDITFSLDEDTQHLFNITGGPLSYSYKITDVKF
HYGIKGYAGSEHKIGNRTFPAEVHMIGYNDDVYKSKPEAEIGAKGIVIIAVFLEIGSKP
NYAMDEIVDHLPGMRKTDYKDMNTVYEQFISSWDLSHLLPNTDQYITYEGSFTQPGCQE
TVTWIIYNKPVYVSENQLDLLRTHGKGDWNYKNTRPVQSINQRAVRTNINFKSKSRFCS
MERKVNYKVNSFLDS

Mytillus coruscus

>McNACR
NWCKVKRRQSPININTNETIYRRYPRLKFENILKRIRNNGHAPHEMFEDEITLCNVPER
PRYNFAQLHVHLGRDEKEGSESIDNKFKPMEAQMVFYDDYEDVAEAKSKRNGLVVISVM
IGRSKNDLTLMEKYEKVRRCEKEFVFEGITPLDVMYDTNSLTSPPCYETVQWVVYKCPI
KVSTKAFKMLLGVRRPLQTTNVVVYRN

>McCA1
HWSDHGQRQSPVDIRTGDVEFASWLTELQFYGYDKIKNNGHTASMAITHSLKLRGAHLN
GDYQLAEMHLHWGFNNSRGSEALNGKKYPAEIHFVHYSTRYGNLSEAMKMPDGLAVIGV
FVPEDTYAFERLANGNNILYGKKANVSNVRMLDFLQRKNSLTTPPCYESVMWFVMADTI
PMSERQLDRFHIRENCRPLQPNGRKVYSN

>McCA2/6
TWCNEGKRQSPINIVPENAMFDQKLASSPLVINYNVMNTGKSVKVQATHSSEIKGGPLT
GTYKLEQFHFHWGANDKKGSETINDKMYASELHLVHYNTKYPNFGAAVDKPDGLAVFGF

FIAKHLGMKELTDTTCSIEGKTCNMACDLDMASLLSDLTSLTTPPLYESVTWVVFKDPI
EMSNDQLALSIVDNYRPPVPGDRTVRAS

>McCA3
HWHEEGVMQSPVAISSEKATYDVSLLQFGFKGFSEMKNNGHTVQVDITSLRTHGGGVTG
GYKPVQFHFHWGADNSKGSEVIDNHHYPMEMHIVHYSDRFQKIEDAIDKPDGLLVLGFF
FPHNIHFAEVFNYENVAKDEIIEPPVVMERLVIDLISLTTPPCYESVVWTVFTQPIKIS
NEQMNDFNLVDDFRPLQQNGRNVYSS

>McCA4a
SVNESMNSTNGLAVLGFLFRHNHKFEIINHHDIQKDDHKSFSLTSLFSTDSLTTPPCYE
SVIWSIFKQHIYISEYQLNQFINNYRNPQPNHRVVTSN

>McCA4b
HWKNEGKQQSPIDIKTDNVKFDPRLRKFNFFDLILIQNNGHTVVDMEDHTIKVTGGGLP
GTYIVEQFHFHWGSVDTRGSEEINGKHFAMEMHVMHSDRFSSVSDAMNRTCGLAVLGFL
VRENPYFEKIISHKEIDINKTVTDKFRLASLFRTDRSLTTPMCYESVIWTIFKEHIYIS
EKQLNKFDMINNYRNPQPNHRVVTTN

>McCA4c
RWGVLGKQQSPIDIRKRDVVYDQNLQLFNLNDLDRFENNGHSVVHLSDKRLRVTGGSLP
GAYNVSQFHFHWGSANSRGSEKINGRHSSMEMHLTHSDKFKNVKEAMNTTNGLVVLGFF
IDYNDEFESIIQNRNIQHKNDSVIIKALSLSDLFQTTRSLTTPPCYESVIWTVFINHIF
ISQYQLQFHINNYRNPQPNGRKVTTN

>McCA4d
QWHTLGKFQSPIDIKTSDVVYDSSLENFNLEELEKCTNHGRSIQGHDIKVTGGSLPGTY
KVEQFHFHWGPNNNVGSETYDGKQVTAELHIVHLDKYSNAGEALKNENGLAVLGYLISH
NKNYDEITDKKNVQQDDIIPQRFKLSSLLSSKRSMTTPPCAPVIWTMFKENIYLSENQL
QEFLIHNCRSPRPDGRIVTSN

>McCA5a
NWTTILRAQSPIDIPDTNAQYDERLVPFVVMGFEKLVNNGHTAQLNIIGDLKVSGGGLS
GTYNTAQLHFHWGSSSNKGSELRNRMAYPLEMHIVNFNSKYNSLVNAAPKPDGLAVLGF
WFAEDNKMFARLVNNTSIQADDEVDTNVLVSDLIIKLDSLTTPPCFESVTWTMFEETIP
ISENQLLFNIVNNFRPVQQNGRRVSRS

>McCA5b
NLLILDIQMLAKLEISGNVLISGGGLPNNYKTAEIHFHWGDTNSVGSERFNGVAAPLEV
QNSRTNPDNPDNPDNPKFSPLLSHSNIIEGNEDSVNDFNLSDLISNLTSMTSPPCLETV
MWSLFPQIIKLSANQLQMFVNNYRPAQPYGRPITKS

>McCA7a
FWPKSGQQQSPISIGVSEVVVNNSLPHFNFDHIKNLENNGHSVQIDLGNNLQLSGGGLP
GTFNAEQFHFHWGSEDKRGSENINGKQYPMEMHVVLFNSKYGMFSNALNKTEGLAVLAF
LFETNHHFDEIISHTKISKDDHALLNTFALDSLFHDAGSLTTPPCYESVIWTIFKNHIV
ISEEQIEKFHISDDYRPIQRNNRVVMTN

>McCA7b
LWPREGLRQSPVDIQTNQAVVDERLGRFDLSDLNMLKNNGHTVDVEGRNNLVVTGGGLP
GPYLVKQFHFHWGSQDNRGSEDINGRFFPMEMHVVTYSQRYRMFEEAKNATGGLAVLSF

LFADNPAFDEIISHNAVQKDDNVAQAFPLGSLFRSTDGLTTPPCYESVIWTIFQEHIRI
SERQINQFHLSDNYRPTQPNGRYVYTN

>McCA8
DWSGVGTKQSPINIVLSSASVDTSLGEFVLTNYDDIKNNGHTIQVDMADDVTLNGGGLG
GPFILAQFHFHWGTSDSTGSETVDGNRSPLEIHFVHYKQSLGDLGTAAGTADGLAVLGF
FFPSDNANLDPFLDASSVAKDQNATLSSPPVIDAIFNTSLGLTTPGCNEVVQWTVFTEK
IRISSTQLAKIGKNYRSTQSNSRTVKIS

>McCA9
FWSCLGTMQSPVNIETNDVVKNSHIRPFYFSGLWKLKNTGHGVPNTQPIIEGGGLPGPH
KLLDFHFHWGGNSQRGSETINGHQFAMEAHFVFLPTTDGPHFAAVIGVLIGSYNKNYEH
IVSKKDVKIGDKAVLDNFPIMNLFETTRSLTTPPCTETVIWTVFEQSICMSEDQISEFI
VDTFRPVQPLQRKVSLS

>McCA10
HWHKLGKQQSPIDITTRSSEHDPKLKDFAIWHDPPVKNNGHSITIETLGPFHVTNGGLP
AVYSTAQFHFHWGHANHQGSELIDSRASPLELHVVNYDSDYQSLPMAMVKPQGLAVLGV
MYEEDNAALEPIIQAKHVEDGHDHEIPAQRIRNFLEDTSSLTTPGCFESVIWTVFHDPM
YISLRQMMAFLVDNFRPVQPNGRKIYRS

>McCA11
HWDKERHHQSPIDIPNNPTLDQSIFMNFNGFENLRNNGHTADSANHSVFLTGNHLGIIF
KTMQFHFHWGKDSTSGSELVKGQSFPLEMHIVNYNYLYKSIQEAMKHSDGLAVIAILFE
EDNEDLRPLIEGEKVKKDSECSINSLSLSKLLKNTRSLTTPGCFESVTWTILSQTQTIS
ERQLDKFIKFNNRPIQGHGRKIYTT

>McCA12
TWNNYGKRQSPIDIGTEYVDYDSSLSPIKINYKPEIVNTGLSVRADIKETSEISGGPLE
GKYRLEQFHFHWGSNSNKGSETLNGYTYASELHLVHWNGKYSSFAEAADKSDGLAVLGF
FIGHEHKELRKLTDGQRIFKDALTKIPDKFDPKHILSDLSSLTTPPFYESVQWIVFADM
IEMSPEQLNLLIDNYRCPLPGERKLRSS

>McCARPa
YWGKLGKHQSPIEIDPSLLLFDPNLTNLHINTANVLETNGLDIDENTQFNITGGPLSYS
YKITDVKFHYGIKGYAGSEKIGNRTFPAEVHMIGYNDEYKSKPEAEIGAKGIVIIAVFL
SKPNYAIEEIVAHPGMRTDYKKFINSWDLSHLLNTDTSFTQPGCQETVTWIIYNKPVYV
SEDQLDLLTNYKNTRPVQSNQRAVRTN

>McCARPb
YWGIHGKYQSPINIDPKILVHDPHMKHLNISKYQIFRNNGHDLNNTNTKPFFITGGPLS
YKYTLYEIKIHFGDHDSIGSESIGGKKFPLEIQLYGYNGENKNISQALSAPHGIAAVSI
LADHDNDYLDILIQAMKVRKGMTTPVLNFFPMEFLVTSVTRPGCMETVTWVILNKPIHV
SHNQLESLENNFRITMPHGRLIRTN

>McCARPc
YWGKAGKYQSPINIDPKSLVHDPNLKRLNVSQNKILLNTGRDLKNTDEKPFIFTGGPLS
YEYTLFEIKIHFGDNNTRGSESIGGKKFPLEIQLYGYNAEHKNSTQALNSPNGIAAISI
LAEEDNINFDALIQSDKVHKGSSIAIDGFSVNAILPLDTSLTQPGCMETVTWIILNKPL
HISFSQLRSLISTNNFRSITSHGRLIRTN
